# P-Rex1 controls phagocytosis and the killing of bacteria by murine neutrophils independently of its catalytic activity

**DOI:** 10.3389/fimmu.2025.1591006

**Published:** 2025-09-30

**Authors:** Priota Islam, Julia Y. Chu, Stephen A. Chetwynd, Rachael Walker, Phillip T. Hawkins, Heidi C. E. Welch

**Affiliations:** ^1^ Signalling Programme, The Babraham Institute, Cambridge, United Kingdom; ^2^ Flow Cytometry Facility, The Babraham Institute, Cambridge, United Kingdom

**Keywords:** PREX1, phagocytosis, NETs, ROS, Rac, Syk, Fc receptors, neutrophils

## Abstract

**Introduction:**

P-Rex1 is a guanine-nucleotide factor for the small GTPase Rac (Rac-GEF) that is known to mediate neutrophil migration and ROS production in response to the activation of GPCRs. These roles of P-Rex1 are assumed to require its activation of Rac.

**Methods:**

Here, we used mice which lack P-Rex1 (*Prex1* ^–/–^) and mice with catalytically inactive P-Rex1 (*Prex1^GD^*) to investigate the importance of the Rac-GEF activity in migration and ROS production. We also studied the requirement for P-Rex1 in degranulation, phagocytosis, NET formation, and the bactericidal activity of neutrophils.

**Results:**

We show that P-Rex1 mediates migration, ROS, and NETs through its Rac-GEF activity, but is dispensable for the degranulation of azurophil, specific and gelatinase granules. Surprisingly, P-Rex1 mediates the clearance of bacteria *in vivo* during septic peritonitis and the killing of bacteria by isolated neutrophils independently of its catalytic Rac-GEF activity. P-Rex1 also mediates the phagocytosis of IgG-opsonized zymosan yeast particles independently of its catalytic Rac-GEF activity. P-Rex1 is required for both integrin-dependent and Fc receptor-dependent phagocytosis, and it mediates the Fc receptor-dependent activation of Rac and Syk. Recently identified adaptor functions of P-Rex1 in GPCR trafficking and glucose uptake do not seem to underlie its GEF-activity independent roles in neutrophils.

**Discussion:**

Together, our study identifies P-Rex1 as a regulator of NET formation and phagocytosis in neutrophils and reveals that P-Rex1 mediates bactericidal activity and phagocytosis independently of its catalytic Rac-GEF activity, through mechanisms not obviously linked to other recently identified adaptor functions.

## Introduction

P-Rex1 (phosphatidylinositol 3,4,5-trisphosphate-dependent Rac exchanger 1, PREX1)^1^ is a guanine-nucleotide exchange factor (GEF) for Rac family small GTPases which is widely expressed and plays important and varied physiological roles in leukocytes, platelets, and endothelial cells during inflammation and infection, in melanoblast migration during development, in the thermogenic potential of brown adipose tissue, in hepatic glucose homeostasis, and in social recognition (1–5). Deregulation of P-Rex1 expression is associated with autism (6) and occurs in many types of cancer, where it can promote tumor initiation, growth, invasion, and metastasis (3, 4, 7, 8).

In the immune system, P-Rex1 is required for a range of pro-inflammatory and host defense responses. In T lymphocytes, P-Rex1 mediates the homeostatic proliferation of naive CD4+ T cells and their differentiation into effector cells (9), but also the TCR-dependent stabilization of inflammatory cytokine transcripts associated with the development of T-cell lymphomas (10). In macrophages, P-Rex1 is required for G protein-coupled receptor (GPCR)-dependent migration and reactive oxygen species (ROS) production (11). Mostly, P-Rex1 has been studied in neutrophils, which are short-lived, terminally differentiated innate immune cells required for immunity against bacterial and fungal infections, for wound healing, and in cancer immunology (12–14). In response to infection, neutrophils are recruited from the blood stream into the infected tissue, where they kill pathogens by a combination of ROS production, degranulation, phagocytosis and the formation of neutrophil extracellular traps (NETs) (12–14). *Prex1* ^–/–^ mouse neutrophils have defects in GPCR-dependent Rac activity, ROS production, F-actin polymerization, and chemokinesis (2, 15–17). Under shear-stress conditions, P-Rex1 mediates the activation of the β2-integrins LFA1 and Mac1 to control neutrophil rolling and crawling along the endothelial vessel wall (18). *In vivo*, *Prex1* ^–/–^ mice show impaired neutrophil and macrophage recruitment during sterile and septic inflammation, and their immunity to bacterial infection is reduced (2, 18–20).

Many types of Rac-GEF are expressed in each cell type (21, 22). In addition to P-Rex1, neutrophils express Vav, Tiam, and Dock family Rac-GEFs, and non-redundant roles have been identified for each of these (23–26). P-Rex1 is unique among them in its synergistic mode of activation by the lipid messenger phosphatidylinositol 3,4,5-trisphosphate (PIP_3_), which is generated by phosphoinositide 3-kinase (PI3K), and by the Gβγ subunits of heterotrimeric G proteins, which are released upon activation of GPCRs (1, 27). This unique mode of regulation makes P-Rex1 an ideal mediator of GPCR signaling, particularly in neutrophils which express Gβγ-dependent PI3Ks and therefore rapidly generate both major P-Rex1 activators upon receptor stimulation (28).

Neutrophils express three isoforms of Rac (Rac1, Rac2, RhoG) with complementary, non-redundant functions (24), although Rac2 is arguably the most important as it is an essential component of the NADPH oxidase enzyme complex which produces ROS (29, 30), as well as being required for neutrophil rolling, spreading, and migration (29, 31), degranulation of azurophil granules (32), phagocytosis of bacteria and IgG-opsonized red blood cells (33, 34), and the formation of NETs (35). Rac1 mediates directional migration (chemotaxis) (36–38), whereas RhoG contributes to F-actin polarity and ROS production (17, 39). *In vivo*, both Rac1 and Rac2 are required for neutrophil recruitment (33, 36, 40–43), and Rac2 mediates antibacterial and antifungal immunity (29, 43). A dominant-negative D57N mutation in Rac2 causes a human immunodeficiency characterized by recurrent infections, defective neutrophil migration, and ROS production. (44). P-Rex1 is primarily a Rac2-GEF but also activates Rac1 and RhoG in neutrophils (1, 2, 15–17).

With two exceptions, all physiological and pathophysiological functions of P-Rex1 are either known or assumed to be mediated through its catalytic Rac-GEF activity. For example, deletion of the Rac-GEF activity abolishes the P-Rex1-dependent migration of neurons and melanoblasts (45–47) and blocks the trafficking of the ionotropic glutamate receptor GluR2 in hippocampal neurons (6). The exceptions are Rac-GEF activity independent functions of P-Rex1 in GPCR trafficking and in glucose homeostasis, which we identified recently. P-Rex1 controls GPCR trafficking by limiting the agonist-induced internalization of the GPCR to maintain high levels of active receptor at the plasma membrane, likely through its direct interaction with the GPCR kinase Grk2 (48). P-Rex1 controls glucose homeostasis independently of its Rac-GEF activity by limiting hepatic glucose uptake, controlling the trafficking of the glucose transporter Glut2, mitochondrial membrane potential, and ATP production in hepatocytes through a mechanism involving the orphan GPCR Gpr21 (5). Here, we used *Prex1^–/–^* mice and mice with catalytically-inactive P-Rex1 (*Prex1^GD^*) to investigate the importance of the Rac-GEF activity of P-Rex1 in neutrophil migration and ROS production, and to study roles of P-Rex1 and its catalytic activity in degranulation, phagocytosis, NET formation, and the bactericidal activity of neutrophils, as well as in the clearance of *E. coli in vivo*.

## Materials and methods

### Mice

*Prex1* ^–/–^ were previously described (2) and were backcrossed at least 10 times to C57BL/6 genetic background. *Prex1* ^GD^ mice with catalytically inactive (GEF-dead) P-Rex1, were generated by the introduction of point mutation N233A in the catalytic DH domain as recently described (5) and were also on C57BL/6 genetic background. Wild type C57BL/6 mice, originally purchased from Charles River (Margate, UK) and imported into the Babraham Institute Small Animal Facility by embryo transfer, were used as controls. Animal breeding and experiments were carried out with approval from the local Animal Welfare Ethical Review Body under the British Home Office Animal Scientific Procedures Act 1986, under Specific Pathogen Free (SPF) conditions, and with quarterly monitoring of sentinels for 62 pathogens, exceeding FELASA guidelines (49). Mice were bred and group-housed (up to 5) in individually-ventilated cages and were fed chow diet (CRM-P, Special Diets Services, Augy, France, 801722) and water *ad libitum*. Mice were used in experiments at the age of 8–14 weeks (typically 10-12). For experiments with isolated cells, mice of both sexes were used. Within experiments, mice were age- and sex-matched between genotypes. For infection with pathogenic *E. coli*, males were used and were housed in individually ventilated isocages in the Babraham Institute biosafety level 2 containment facility.

### Peritonitis

Pathogenic *E. coli* O18:K1 bacteria were used to induce septic peritonitis essentially as described (20, 50). The bacteria were grown in a biosafety level 2 facility to mid-log phase in Luria broth (LB) at 37°C, 5% CO_2_, pelleted, snap-frozen in PBS/20% glycerol and stored in aliquots at −80°C. Titers were determined by CFU count on LB agar plates. Prior to infection, a fresh aliquot of bacterial stock was thawed, washed in ice-cold Dulbecco’s phosphate buffered saline (DPBS) without Ca^2+^ and Mg^2+^ (DPBS^–^, Sigma, D8537) by centrifugation at 10,000 × g for 2 min at 4 °C, resuspended at 5 × 10^4^ bacteria/ml in ice-cold DPBS^–^, kept on ice and used within 1.5 h. To induce septic peritonitis, mice in a biosafety level 2 containment facility were injected *i.p.* with 200 µl of the *E. coli* suspension (1 × 10^4^ bacteria per animal), or were mock-treated with DPBS^–^, before being returned to their home cages with food and water *ad libitum*. 3 h later, mice were euthanized by CO_2_ asphyxiation, death confirmed by pithing, and peritoneal lavages performed by *i.p.* injection and aspiration of 8 ml DPBS^–^, 5 mM EDTA. A second lavage was performed, pooled with the first, and samples were stored on ice. An aliquot of the lavage fluid was serially diluted in ice-cold DPBS^–^, plated onto LB agar plates, cultured overnight at 37°C, 5% CO_2_, and CFU were counted on plates of comparable density (20–200 bacteria). The remaining lavage cells were pelleted at 450 × g for 10 min at 4°C, erythrocytes lysed by resuspending cells in 1 ml Geye’s solution (130 mM NH_4_Cl, 5 mM KCl, 780 µM Na_2_HPO_4_, 176 µM KH_2_PO_4_, 5.5 mM glucose, 1 mM MgCl_2_, 280 µM MgSO_4_, 1.54 mM CaCl_2_, 13.4 mM NaHCO_3_) and incubation at RT for 150 s, prior to the addition of 10 ml DPBS with Ca^2+^ and Mg^2+^ (DPBS^++^, Sigma, D8662) supplemented with 0.1% glucose (Sigma, G8769) and 4 mM NaHCO_3_ (Sigma, S8761), all of tissue culture grade (DPBS^++++^). Leukocytes were centrifuged again and resuspended in 1 ml fixation buffer (Biolegend, 420801), incubated for 20 min at RT, washed again, and resuspended in 1 ml DPBS^++++^. Aliquots of fixed cells were stained with AF647-Cd11b (clone M1/70, BD Pharmingen, 557686, 1:1000), BV421-Ly6G (clone 1A8, BioLegend, 127628, 1:500) and BV510-CD45 (clone 30-F11, BD Biosciences, 536891, 1:500) antibodies in PBS with Fc block (clone 2.4G2, BD Biosciences, 553141, 1:1000) for 20 min on ice in the dark, washed in DPBS^–^, 5 mM EDTA, and resuspended in 300 µl DPBS^–^, 5 mM EDTA. Flow cytometry was carried out in a BioRad ZE5 flow cytometer, and FlowJo was used for data analysis. Neutrophils were identified by Cd11b^hi^, Ly6G^hi^ staining, macrophages by CD11b^hi^ and Ly6G^low^ staining, and total leukocytes by CD45^hi^ staining. Leukocytes were enumerated by considering the lavage volume recovered.

### Neutrophil isolation

Mature primary neutrophils were freshly purified from mouse bone marrow each day using a Percoll^PLUS^ gradient at 4°C and endotoxin-free reagents, as previously described (25, 50, 51). Bone marrow cells were flushed from mouse femurs, tibias, and pelvic bones using ice-cold Hank’s balanced salt solution without Ca^2+^ or Mg^2+^ (HBSS^–^, Sigma H6648) supplemented with 15 mM HEPES, pH 7.4 (RT) (Sigma, H3784) and 0.25% fatty acid-free BSA (HBSS^–++^, Sigma, A8806). The bone marrow cells were triturated and filtered through 40 μm cell strainers. 58% isotonic Percoll^PLUS^ (GE Healthcare, 17544501) in HBSS^–++^ was added as an underlayer, and samples were centrifuged at 1620 × g without brake for 30 min at 4°C. The lower 3 ml were resuspended in 40 ml HBSS^–++^ and centrifuged at 326 × g for 10 min at 4°C. Erythrocytes were lysed in Geye’s solution for 3 min on ice. 10 volumes of ice-cold HBSS^–++^ were added, and the cells were centrifuged again. Neutrophils were resuspended in ice-cold DPBS^++++^ and kept on ice. Aliquots were counted using a hemocytometer, and purity was assessed by Kwik-Diff (Thermo Scientific Shandon, 9990700) staining of cytospins. Preparations were typically 90-95% pure and yielded ~1.6 × 10^7^ neutrophils per mouse. Neutrophils were sedimented, resuspended in the buffer appropriate for the subsequent assay, and kept on ice until use.

### Killing of bacteria *in vitro*

The ability of isolated neutrophils to kill *Staphylococcus aureus* was measured essentially as described (20). *S. aureus* (Wood 46) was stored at -80°C as glycerol stocks. Prior to experiments, bacteria were cultured in LB at 37°C to logarithmic growth, enumerated by OD_600,_ sedimented, and opsonized with 50% mouse serum in DPBS^++++^ for 30 min at 37°C, before ice-cold DPBS^++++^ was added to give 1.5 × 10^8^ bacteria/ml in 10% mouse serum, and bacteria were kept on ice. Purified bone marrow-derived neutrophils at 2.5 × 10^7^/ml in DPBS^++++^ were primed with 20 ng/ml murine tumor necrosis factor α (TNFα, R&D Systems, 410-MT-010) and 50 ng/ml granulocyte-macrophage colony-stimulating factor (GM-CSF, Peprotech, 315-03) for 45 min at 37°C. For negative controls, neutrophils were heat-killed for 45 min at 55°C and then kept on ice. Opsonized bacteria and heat-killed neutrophils were prewarmed to 37°C for 3 min prior to the assay. 50 µl serum-opsonized *S. aureus* (7.5 × 10^6^/sample) were incubated with 200 µl primed neutrophils (5 × 10^6^/sample) for 90 min at 37°C, at a ratio of 1.5 bacteria per neutrophil. In some experiments, 10 μM diphenyleneiodonium (DPI; Sigma D2926), or vehicle control (1% DMSO), were included during priming and killing assay. After the incubation, 50 µl aliquots were added to 950 µl ice-cold LB, 0.05% saponin, and samples were incubated on ice for 10 min with frequent vortexing. Serial dilutions were plated onto LB-agar and incubated overnight at 37°C to enumerate bacterial colonies. Bacterial CFU in samples with live neutrophils were expressed as % of samples with heat-killed neutrophils.

### Chemotaxis

Transwell chemotaxis assays were done essentially as described (25, 26, 50) using 3 µM-pore polycarbonate filters (Millipore, Millicell-PC, PITP01250) in ultra-low cluster 24-well tissue culture plates (Costar, 3473). Bone marrow was flushed into HBSS with Ca^2+^ and Mg^2+^ (Sigma, H8264), supplemented with 0.25% fatty acid-free BSA, and 15 mM Hepes, pH 7.5 at 37°C, all endotoxin-free (HBSS^++++^), triturated, filtered through a 40 µm nylon cell strainer, counted by hemocytometer and adjusted to 5 × 10^6^/ml. Cells were primed with 20 ng/ml TNFα and 50 ng/ml GM-CSF for 45 min at 37°C, pipetted into transwell filters (400 µl/filter) in a 24-well plate containing HBSS^++++^ (600 µl/well) in the presence or absence of 3 nM complement factor C5a anaphylatoxin (C5a, R&D Systems, 2037-C5-025), and incubated for 40 min at 37°C. Cells remaining in the transwell were removed and replaced with 400 µl ice-cold HBSS^–++^ containing 3 mM EDTA. 60 µl HBSS^–++^ containing 30 mM EDTA was added to the bottom well, and plates were incubated on iced metal trays for 15 min to detach cells. Transmigrated cells were collected from the bottom well and, in parallel to control cells that had not undergone chemotaxis, were centrifuged at 10,000 × g for 30 s and resuspended in ice-cold HBSS^–++^. Cells were stained with FITC-Ly6G (clone 1A8, BioLegend, 127606, 1:800) and AF647-Cd11b (clone M1/70, BioLegend, 101218, 1:800) antibodies in HBSS^–++^ containing Fc block (1:1000) and were analyzed using a BioRad ZE5 flow cytometer alongside Spherotech ACBP-50–10 standard beads. Neutrophils were identified by their Ly6G^hi^/Cd11b^hi^ staining. Transmigrated neutrophils were compared to total neutrophils in control samples.

### ROS

ROS production was measured by luminol chemiluminescence assay in a Berthold MicroLumat Plus luminometer (Berthold Technologies), essentially as previously described (20, 25, 50). To measure ROS production in response to soluble stimuli, purified bone-marrow derived neutrophils were resuspended at 5 × 10^6^ cells/ml in ice-cold DPBS^++++^ and primed with 1 μg/ml lipopolysaccharide (*E. coli* LPS, Sigma, L3024) for 90 min at 37°C with occasional flicking to prevent settling. Unprimed neutrophils were kept on ice and prewarmed to 37°C for 3 min prior to the assay. Stimuli f-Met-Leu-Phe (fMLP, Sigma, F3506) and phorbol 12-myristate 13-acetate (PMA, Sigma, P1585) were prepared as 2.5× stocks in DPBS^++++^. Prior to the assay, an equal volume of prewarmed Detect 1 [DPBS^++++^, 53 units/ml horseradish peroxidase (HRP, Sigma, P8375), 400 µM luminol (Sigma-Aldrich, 123072)] was added to the prewarmed, primed or unprimed neutrophils. The neutrophil/Detect mix was incubated for 3 min at 37°C, before 150 µl/well were dispensed into a prewarmed 96-well luminometer plate. 100 µl of prewarmed 2.5× stimulus in DPBS^++++^, or DPBS^++++^ for mock treatment, was added either by automatic injection port (fMLP) or manually (PMA), and real-time ROS production was recorded at 37°C. Final assay concentrations were 1.5 × 10^6^ neutrophils/ml, 120 luminol, 16 units/ml HRP, and 3 µM fMLP or 500 nM PMA. ROS production was quantified by integrating the area under the curve (AUC) of the ROS response over 2 min for fMLP or 10 min for PMA.

To measure intracellular ROS production, neutrophils at 1 × 10^7^ cells/ml in DPBS^++++^ were primed with 20 ng/ml TNFα and 50 ng/ml GM-CSF for 45 min at 37°C, in the presence of 50 units/ml superoxide dismutase (SOD, Sigma, S9697) and 2000 units/ml catalase (Sigma, C100) to scavenge extracellular ROS (52). The primed cells were diluted to 5 × 10^6^/ml with prewarmed DPBS^++++^, and an equal volume of prewarmed Detect 2 (DPBS^++++^, 400 µM luminol, 165 units/ml SOD, 6600 units/ml catalase) was added. The neutrophil/Detect mix was incubated for 3 min at 37°C, before 150 µl/well were dispensed into a prewarmed 96-well luminometer plate, and 100 µl of prewarmed 2.5× stimulus was added using a multichannel pipette. Stimuli were serum-opsonized *S. aureus*, prepared as described above, but washed after opsonization to remove any remaining serum (ratio of bacteria/neutrophil 10:1), and IgG-opsonized zymosan yeast particles (5:1) or IgG-opsonized latex beads (10:1), prepared as described below for the phagocytosis assays, except that the latex beads used here were unlabeled to avoid interference with the luminol signal. Beads were either 3 μm (PolyBead Microspheres, 3.00 μm, PolySciences, 19814) or 2 μm (PolyBead Microspheres, 2.00 μm, PolySciences, 17134) in diameter. Final assay concentrations were 1.5 × 10^6^ neutrophils/ml, 120 µM luminol, 50 units/ml SOD, 2000 units/ml catalase, and 1.5 × 10^7^ serum-opsonized *S. aureus*/ml, 7.5 × 10^6^ IgG-opsonized zymosan/ml, or 1.5 × 10^7^ IgG-opsonized latex beads/ml. Intracellular ROS production was quantified as the AUC of the ROS response over 60 min.

To assess cell shape changes induced by particles, neutrophils were recovered after the intracellular ROS assays, spun down for 2 min at 2000 × g, resuspended in 200 μl 4% paraformaldehyde in PBS, 1 mM EGTA, 0.5 mM MgCl_2_, and fixed for 15 min at RT. 1 ml PBS was added, and cells were sedimented at 10000 × g for 1 min before being resuspended in 200 μl PBS. Cells were analyzed by brightfield imaging on an Amnis ImageStream^x^ MkII imaging flow cytometer (Cytek Biosicences), acquiring up to 5000 cells per sample over 10 min. Cells in focus in the brightfield images were assessed for their median circularity using the ImageStream software, where decreasing values mean decreasing circularity.

### Degranulation

Degranulation of myeloperoxidase (MPO) from azurophil granules, lactoferrin from specific granules, and gelatinase (metalloproteinase 9, Mmp9) from gelatinase granules of neutrophils stimulated with *E. coli* was measured by western blotting. *E. coli* (DH5α) were cultured in LB at 37°C to logarithmic growth on the day of the experiment, enumerated by OD_600,_ washed in DPBS^++++^, opsonized with 10% mouse serum in DPBS^++++^ for 15 min at 37 °C, washed twice in DPBS^++++^, resuspended in ice-cold DPBS^++++^ at 2.5 × 10^9^/ml, and kept on ice. Purified bone marrow-derived neutrophils were resuspended at 1.82 × 10^7^/ml in DPBS^++++^, and 55 µl (1 × 10^6^ cells) were stimulated with 5 µl *E. coli* at a ratio of 12.5 bacteria per neutrophil at 37 °C for various periods of time up to 3 h. Neutrophils were sedimented at 326 × g for 10 min at 4°C, and the supernatant was recovered and cleared at 10,000 × g for 1 min. As a control, pellets of neutrophils prior to stimulation were collected and washed in DPBS^++++^. The cleared supernatants and washed pellets were resuspended in boiling 1.3× Laemmli sample buffer and boiled for 5 min before snap-freezing in liquid nitrogen. Samples were analyzed by SDS-PAGE and western blotting with MPO (R&D Systems, AF3667, 1:3000), lactoferrin (BosterBio, A00633-1, 1:2000) and gelatinase (Abcam, ab38898, 1:1000) antibodies, with quantification by Fiji (ImageJ) densitometry. The percentage of secreted protein was calculated as % of the total protein in the 0’ control cell pellet.

### NET formation

NET formation in response to *S. aureus* was assayed essentially as described (20, 25). *S. aureus* were cultured in LB to logarithmic growth on the day of the experiment, sedimented for 2 min at 12,000 × g, washed in Dulbecco’s modified Eagle medium (DMEM) with Ca^2+^, Mg^2+^, and 4.5 g/l glucose (Thermo Fisher Scientific, 31053) supplemented with 10 mM Hepes, pH 7.4 (DMEM^++++^), opsonized with 10% mouse serum for 30 min at 37°C, washed twice in DMEM^++++^, and resuspended at 5 × 10^7^ bacteria/ml in DMEM^++++^. Purified neutrophils were resuspended at 4 × 10^5^ cells/ml in DMEM^++++^ containing 10% heat-inactivated FBS, and 250 μl cells were seeded into each well of an 8-well chamber slide (μ-slide 8 well, 80826, ibidi) and allowed to adhere for 30 min at 37°C, 5% CO_2_. Cells were stimulated with serum-opsonized *S. aureus* at a ratio of 10 bacteria per neutrophil or mock-stimulated with DMEM^++++^ for various periods of time up to 3 h. 15 min before the end of the incubation, the non-cell permeable DNA dye Sytox Green (Thermo Fisher Scientific, S7020, 0.1 μM) and the cell-permeable DNA dye Hoechst 33342 (Thermo Fisher Scientific, 62294) were added, and samples were live-imaged using a Nikon Eclipse Ti-E widefield system. Images were analyzed by ImageJ, using phase contrast and DAPI staining to determine total cell numbers and Sytox Green signal to enumerate cells with NETs.

To assay NETs formation in a different manner, we determined the release of DNA, citrullinated histone 3 (CitH3), and MPO by western blotting and NanoDrop, essentially as previously described (50). Neutrophils were resuspended at 2 x 10^7^/ml in DPBS^++++^, and 50 μl (1 x 10^6^ cells/sample) were aliquoted into 2 ml Eppendorf tubes. *S. aureus*, grown and opsonized with mouse serum as described here-above, were centrifuged for 1 min at 10,000 × g to remove the serum, and resuspended at 2.5 × 10^8^ bacteria/ml in DPBS^++++^. 50 μl bacterial suspension (1.25 × 10^7^ bacteria/sample; ratio of 12.5:1) were added to the cells, and samples were incubated for 3 h at 37°C without disturbing them. Alternatively, cells were mock-stimulated for the same time in DPBS^++++^. A zero-time control of mock-stimulated cells was also prepared, by centrifuging cells for 1 min at 10000 × g, adding boiling 1.3× sample buffer to the pellet, boiling, and then snap-freezing the sample in liquid N_2_. After the 3 h incubation, the remaining samples were centrifuged at 326 × g for 10 min at 4°C, the supernatant was transferred into fresh tubes, spun for 1 min at 10000 × g to clear it of any remaining cells, and 10 μl of the cleared supernatant were removed for DNA concentration analysis by NanoDrop spectrophotometer. Boiling 4× sample buffer was added to the remaining cleared supernatant, and samples were boiled and then snap-frozen in liquid N_2_. Samples were analyzed by western blotting for MPO (R&D Biosystems, AF3667, 1:3000) and CitH3 (Abcam, AB5103, 1:4000) on the same membrane cut horizontally for direct comparison.

### Phagocytosis

Phagocytosis of IgG-opsonized zymosan yeast particles was assayed essentially as described (20, 26). Zymosan A particles (Molecular Probes, Z2894) were opsonized using IgG from mouse serum (Sigma-Aldrich, I8765) for 1 h at 37°C, on the day of the experiment, washed in DPBS^++++^, stored on ice at 2.5 × 10^7^ particles/ml in DPBS^++++^, and prewarmed for 5 min before use. Purified neutrophils were resuspended at 5 × 10^6^ cells/ml in DPBS^++++^, and 100 μl were prewarmed to 37°C for 45 min and allowed to adhere to sterile glass coverslips in a 24-well plate for 15 min at 37°C, 5% CO_2_, before 100 μl IgG-opsonized zymosan particles were added at a ratio of 5 particles per neutrophil and samples incubated for a further 30 min at 37°C, 5% CO_2_. Samples were fixed in 4% paraformaldehyde, PBS for 15 min at RT, washed twice in PBS, permeabilized in PBS/0.1% Triton X-100 for 10 min at RT, and washed twice in PBS. Samples were stained with anti-mouse IgG-AF568 (ThermoFisher, A11036, 1:100), FITC-Gr1 antibody (Thermo Fisher Scientific, RM3001, 1:500), and Hoechst 33342 DNA dye (Thermo Fisher Scientific, 62294, 1:400), in PBS containing Fc block (1:100), for 40 min at RT, followed by three washes in PBS. Samples were mounted in ProLong Gold Antifade (Thermo Fisher Scientific, P36930) and imaged using a Zeiss Axio Imager D2 widefield system. Zymosan internalization was quantified using ImageJ image analysis to determine the percentage of neutrophils that phagocytosed at least one at least one particle, the mean number of phagocytosed particles in all neutrophils, and the mean number of phagocytosed particles per phagocytosing neutrophils.

Phagocytosis of latex beads (Sigma-Aldrich, L3030, red fluorescent, 2.0 μm mean diameter) was assayed in a similar manner, except that the latex beads were opsonized using a range of different methods, or were left unopsonized, and were used at a ratio of 10 beads per neutrophil, and neutrophils were primed with 20 ng/ml TNFα and 50 ng/ml GM-CSF for 45 min at 37°C, or mock-primed, prior to plating, and the time of stimulation was extended to 60 min. For opsonization with IgG, latex-beads were washed in DPBS^++++^, incubated with IgG from mouse serum for 1 h at 37°C, washed, and resuspended at 5 x 10^7^ beads/ml. For serum opsonization, latex beads were incubated with 50% mouse serum in DPBS^++++^ for 60 min at 37°C, washed, and resuspended at 5 x 10^7^ beads/ml. For opsonization with complement-inactivated mouse serum, the serum was heat-inactivated for 1 h at 56°C prior to use (53). For opsonization with immunoglobulin-deficient serum, serum from *Rag2^–/–^Il2rγc^–/–^* (*Rag2^–/–^*) mice (53) was used. For opsonization with complement and immunoglobulin-deficient serum, *Rag2^–/–^* serum was heat-inactivated prior to use.

### Erk, p38 Mapk and Syk signaling

IgG-opsonized latex beads were prepared as described in the phagocytosis section and prewarmed for 5 min prior to use. Purified neutrophils at 1 × 10^7^/ml in DPBS^++++^ were primed with 20 ng/ml TNFα and 50 ng/ml GM-CSF for 45 min at 37°C, and 150 µl aliquots were then incubated for 30 min at 37°C with IgG-opsonized latex beads added at a ratio of 10 particles per neutrophil for varying periods of time towards the end of that incubation. The reaction was stopped with 1 ml ice-cold DBPS^++++^, and the cells were sedimented at 10000 × g for 30 s at 4°C. Cells were lysed in 150 µl ice-cold RIPA buffer (30 mM Hepes, pH 7.4, 150 mM NaCl, 1% Nonidet P-40, 0.5% deoxycholate, 0.1% SDS, 5 mM EGTA, 4 mM EDTA) supplemented with 1 mM DTT, protease inhibitors (100 μM PMSF, and 25 μg/ml each of leupeptin, pepstatin-A, aprotinin and antipain), and phosphatase inhibitors (50 mM NaF, 10 mM β-glycero-phosphate) for 5 min on ice with frequent vortexing. Debris was sedimented at 12,000 x g for 4 min at 4°C, and the supernatant transferred into fresh tubes. 75 µl boiling 4× SDS-sample buffer were added and samples boiled for 10 min before snap-freezing in liquid nitrogen. Samples were western blotted using primary antibodies from Cell Signaling Technology (London, UK), comprising phospho-p38 Mapk (9211, 1:500), p38 Mapk Thr180/Tyr182 (9212, 1:1000), phospho-Erk1/2 Thr202/Tyr204 (4370, 1:4000), Erk1/2 (9102, 1:2000), phospho-Syk (2710, 1:1000), Syk (2712, 1:1000) and secondary goat anti-rabbit IgG (Bio-Rad, 1706515, 1:3000). Detection was done with Clarity ECL reagent (Bio-Rad, 1705060, 1:3000). Blots for phosphorylated proteins were processed first, then stripped in Restore PLUS Western Blot Stripping Buffer (Thermo Fisher, 46430), and reprobed for the total proteins. Coomassie staining was used to control for protein loading. The activities of Erk, p38 Mapk and Syk were quantified using Fiji densitometry, dividing the phospho-signals for each protein by the coomassie signals over the whole lane.

### Rac activity

Rac activity was assessed by Pak-CRIB pull down, essentially as described (20, 25). GST-Pak-CRIB bait was purified from bacterial culture and stored in GST-FISH buffer (10% glycerol, 50 mM Tris pH 7.4, 100 mM NaCl, 1% NP-40, 2 mM MgCl_2_, 2 mM DTT, 100 µM PMSF, and 10 µg/ml each of leupeptin, pepstatin A, aprotinin and antipain) at 4°C for up to one week. For measuring Rac activity in response to fMLP, purified neutrophils at 1 × 10^7^/ml in DPBS^++++^ were pre-warmed for 3 min at 37°C. 200 µl aliquots were stimulated with 10 µM fMLP in DPBS^++++^ for 0, 5 or 10 s. The reaction was stopped by the addition of 1 ml of ice-cold GST-FISH buffer containing 1.2% NP-40 (for final 1% NP-40), and cells were lysed by incubation on ice for 2 min with frequent vortexing. Samples were centrifuged at 12,000 × g for 3 min at 2°C to sediment debris, and the supernatant was transferred into fresh precooled tubes. 2% were kept as a total lysate control. The rest was incubated with GST-Pak-CRIB beads by end-over-end rotation for 15 min on ice. Samples were washed 5 times in GST-FISH buffer before the addition of boiling 1.3× SDS-PAGE sample buffer and boiling the samples for 5 min. For total lysate samples, boiling 4× SDS-PAGE sample buffer was added to final 1.3×, and samples were boiled for 5 min. GTP-Rac1, GTP-Rac2 and total Rac1 and Rac2 were quantified by western blotting with Rac1 (clone 23A8, Millipore, 05-389, 1:3000) and Rac2 Millipore, 07-604, 1:5,000) antibodies. Fiji densitometry was used to quantify Rac1 and Rac2 activity, with normalizing the active, GTP-bound Rac signal to the total Rac protein.

To measure Rac2 activation in response to IgG-opsonized latex beads, prepared as described in the phagocytosis section, were prewarmed at 37°C for 5 min prior to use. Neutrophils at 1 × 10^7^/ml in DPBS^++++^ were primed with 20 ng/ml TNFα and 50 ng/ml GM-CSF for 45 min at 37°C and then incubated for 30 min at 37°C with IgG-opsonized beads added at a ratio of 10 beads per neutrophil for varying periods of time towards the end of that incubation. Total lysates were prepared and active, GTP-bound Rac2 was isolated and quantified by western blotting as described here above.

### Cell surface receptor levels

The cell surface levels of neutrophil phagocytosis receptors were measured essentially as described (50). Bone marrow cells were flushed with ice-cold HBSS^–++^, filtered through 40 μm cell strainers, counted, pelleted at 326 × g for 10 min at 4°C, and resuspended in ice-cold DPBS^++++^ at 4 × 10^7^ cells/ml. 125 μl of the cells were either kept on ice, or were primed with 20 ng/ml TNFα and 50 ng/ml GM-CSF, or mock-primed in DPBS^++++^, for 45 min at 37°C. Cells were sedimented at 10,000 × g for 30 s at 4°C, resuspended in Fc block (1:1000) for CD11b/Mac1 or in DPBS^++++^ for CD16/FcγRIII and CD32/FcγRII, and were incubated on ice for 15 min. Cells were sedimented at 10,000 × g for 30 s, resuspended in ice-cold DPBS^++++^ containing fixable viability dye (eBioscience, 65-0865-14, 1:1000), antibodies for neutrophil markers Ly6G (Ly6G-BV510, clone 1A8, BioLegend, 127627, 1:500) and Mac1 (CD11b-AF647, clone M1/70, BD Biosciences, 557686, 1:1000), and antibodies for FcγRIII (CD16-PE, clone S17014E, BioLegend, 158003, 1:100) or FcγRII (CD32-FITC, clone S17012B, BioLegend, 156408, 1:100), and were incubated on ice for 30 min. Cells were washed in ice-cold HBSS^–++^, 1 mM EDTA, resuspended in 300 μl ice-cold HBSS^–++^, 1 mM EDTA, and kept on ice. Flow cytometry was performed using a BioRad ZE5 flow cytometer, recording 20,000 neutrophils per sample. Neutrophils were identified by Ly6G^hi^, CD11b^hi^ staining, and the mean fluorescence intensity (mfi) of receptor levels on the neutrophil surface was quantified using FlowJo.

The agonist-induced internalization of the GPCRs C5aR1 and CXCR4 was measured essentially as described (51). Bone marrow cells prepared as here-above were resuspended in ice-cold DPBS^++++^ at 4 × 10^7^ cells/ml, and 125 μl of cells were incubated for 30 min at 37°C with 50 nM C5a or 100 nM stromal cell-derived factor 1α (SDF1α, Peprotech, 250-20A) added for various periods of time towards the end of that incubation. Cells were sedimented at 10,000 × g for 30 s at 4°C, resuspended in DPBS^++++^/Fc block, and incubated on ice for 15 min. Cells were sedimented at 10,000 × g for 30 s, resuspended in ice-cold DPBS^++++^/Fc block, fixable viability dye (eF780) (Invitrogen, 65-0865-14, 1:1000), antibodies for neutrophil markers Ly6G (Ly6G-BV421, clone 1A8, BioLegend, 127627, 1:500) and Mac1 (CD11b-AF647, clone M1/70, BioLegend, 101218, 1:1000) and PE-labelled antibodies for C5aR1 (Abcam, ab53434, 1:60) or CXCR4 (Invitrogen, 12-9991-82, 1:60), and were stained for 30 min on ice. Cells were sedimented at 10,000 × g for 30 s at 4°C, and pellets were resuspended in 400 μl HBSS^–++^/1 mM EDTA and analyzed using a using a BioRad ZE5 flow cytometer. Neutrophils were identified by their Ly6G^hi^/Cd11b^hi^ staining, and C5aR1 and CXCR4 cell surface levels were determined from the PE signal intensity using FlowJo.

### Glucose uptake

Glucose uptake was measured essentially as described (5). Purified neutrophils were resuspended in Krebs Ringer buffer (KRB; 12 mM Hepes (pH7.4), 121 mM NaCl, 4.9 mM KCl, 1.2 mM MgSO_4_, 0.33 mM CaCl_2_) supplemented with 0.1% fatty-acid free (FAF)-BSA (Sigma, A6003) and incubated for 90 min at 37°C, 5% CO_2_. 200 nM insulin (Actrapid, Novo Nordisk) was added for 30 min to give a final insulin concentration of 100 nM, or cells were mock-stimulated in KRB/0.1%FAF-BSA. 150 μM 2-deoxy glucose (DOG, Cayman Chemical Company, 14325) was added for a further 30 min to give a 50 μM final concentration, together with 0.25 μCi H^3^-2-DOG (Perkin Elmer NET328A250UC) per sample. Cells were washed three times in ice-cold KRB at 10000 × g, 30 s, 4°C, and lysed in 250 μl RIPA buffer for 5 min on ice. Samples were mixed with Ultima Gold scintillation fluid (Perkin Elmer, 6013326) and analyzed in a Tri-Carb 2900TR scintillation counter (Perkin Elmer).

### Data collection and statistical analysis

Sample size was determined using power calculations to yield 80% power, based on results of pilot experiments and on previously published data as referenced. Animals were selected for experiments as described under ‘Mice’ according to genotype, sex, and age. Within these criteria, mice were selected at random by the staff of the Biological Support Unit. Experiments were performed at least three times. Statistical analysis and plotting of graphs were done in GraphPad Prism 10. Data were tested for normality of distribution to determine if parametric or non-parametric methods of analysis were appropriate. Where warranted, data were log-transformed or square-root transformed prior to analysis. Outliers were identified using Tukey’s test and were excluded from the analysis. Otherwise, only samples with known technical errors were excluded from the analysis. For comparison of two groups, paired Student’s t-test was used. For comparison of multiple groups, one-way or two-way ANOVA were used, as appropriate, with repeated measures followed by the *post-hoc* multiple comparisons correction test recommended by GraphPad. Statistical analysis was done on raw data. Where it was necessary to compare normalized data, the normalized values were excluded from the analysis. Parameters with values of p ≤ 0.05 were considered to differ significantly. Results are presented as mean ± standard error of the mean (SEM). In the figures, p-values in black denote significant differences, p-values in grey are non-significant. Sample size and the numbers of independent experiments are detailed in figure legends.

## Results

### P-Rex1 deficiency or loss of its catalytic Rac-GEF activity do not affect neutrophil development

Studies on *Prex1* ^–/–^ mice have shown that P-Rex1 is required for neutrophil adhesion, migration, and ROS production (24), and these functions are assumed to be mediated through the catalytic Rac-GEF activity. We recently generated a *Prex1* ^GD^ mouse strain where endogenous P-Rex1 is rendered catalytically inactive by an N233A point mutation in the catalytic DH domain and used this strain to show that P-Rex1 plays roles in glucose homeostasis which are independent of its catalytic Rac-GEF activity (5). Here, we used *Prex1* ^–/–^ and *Prex1* ^GD^ mice to further study P-Rex1 functions in neutrophils and assess which of these require its Rac-GEF activity.

We previously reported that neutrophil development is normal in *Prex1* ^–/–^ mice (2). The same is true for *Prex1* ^GD^ mice, which have normal numbers of mature bone-marrow derived neutrophils that express the characteristic CD11b^hi^, Ly6G^hi^ marker proteins and have the doughnut-shaped nuclear morphology characteristic of mature mouse neutrophils (**Supplementary Figure 1**). As neutrophil development was normal, any differences between wild type, *Prex1* ^–/–^, and *Prex1* ^GD^ neutrophils are therefore caused by effects of P-Rex1 on the functional competency of neutrophils.

### P-Rex1 is required for innate immunity during acute *E. coli* infection independently of its catalytic Rac-GEF activity

**Figure 1:**
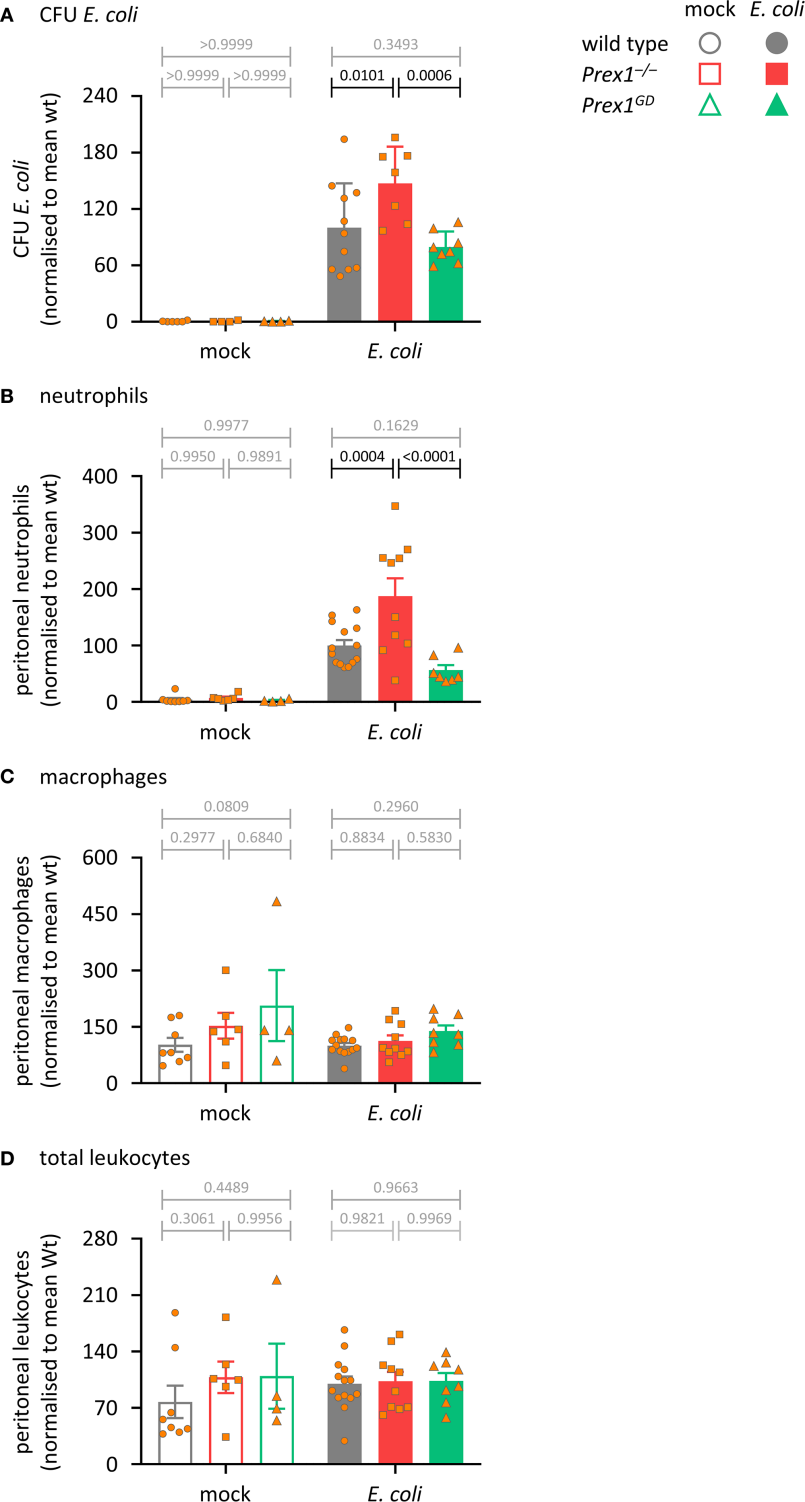
P-Rex1 is required for immunity against acute peritoneal *E. coli* infection independently of its catalytic Rac-GEF activity. *Prex1^–/–^* mice (red squares), *Prex1^GD^* mice with catalytically inactive (GEF-dead) P-Rex1 (green triangles) and C57BL/6 wild type mice (grey circles) were infected *i.p.* with 1 x 10^4^ CFU pathogenic *E coli* O18:K1 (closed symbols), or mock-infected (open symbols), culled humanely 3 h later, and peritoneal lavages performed. **(A)** Peritoneal lavages were assessed for bacterial burden by bacterial culture and quantification of CFU. **(B-D)** Lavage neutrophils **(B)**, macrophages **(C)**, and total leukocytes **(D)** were quantified by flow cytometry. Data are mean ± SEM of mice pooled from 3–4 independent experiments, with 1–2 mock-infected and 2–3 infected mice from each genotype per experiment. Each symbol represents one mouse. Statistics are two-way ANOVA with Sidak’s multiple comparisons test on raw data; black p-values are significant, grey p-values non-significant.

P-Rex1 is required for the clearance of *S. pneumoniae* bacteria during acute pulmonary infection (20). To test whether the P-Rex1-depedendent clearance of bacteria requires the catalytic Rac-GEF activity, we compared here wild type, *Prex1* ^–/–^, and *Prex1* ^GD^ mice in an acute peritonitis model induced by infection with 10^4^ CFU of the pathogenic *E. coli* strain O18:K1. *Prex1* ^–/–^ mice showed a reduced ability to clear the *E. coli* bacteria from the infected peritoneum, despite increased neutrophil recruitment and normal peritoneal macrophage and total leukocyte numbers (**Figure 1**), confirming that P-Rex1 is required for innate antibacterial immunity. In contrast, the ability of *Prex1* ^GD^ mice to clear bacteria was the same as that of wild type mice (**Figure 1**). These data suggested that P-Rex1 mediates the clearance of *E. coli in vivo* independently of its catalytic Rac-GEF activity. The finding that neutrophil recruitment was not reduced in *Prex1* ^–/–^ mice suggested that impaired neutrophil effector responses may contribute to their reduced immunity.

### P-Rex1 mediates the killing of *S. aureus* by neutrophils independently of its catalytic Rac-GEF activity, whereas chemotaxis, ROS, and NETs formation do require its Rac-GEF activity

**Figure 2:**
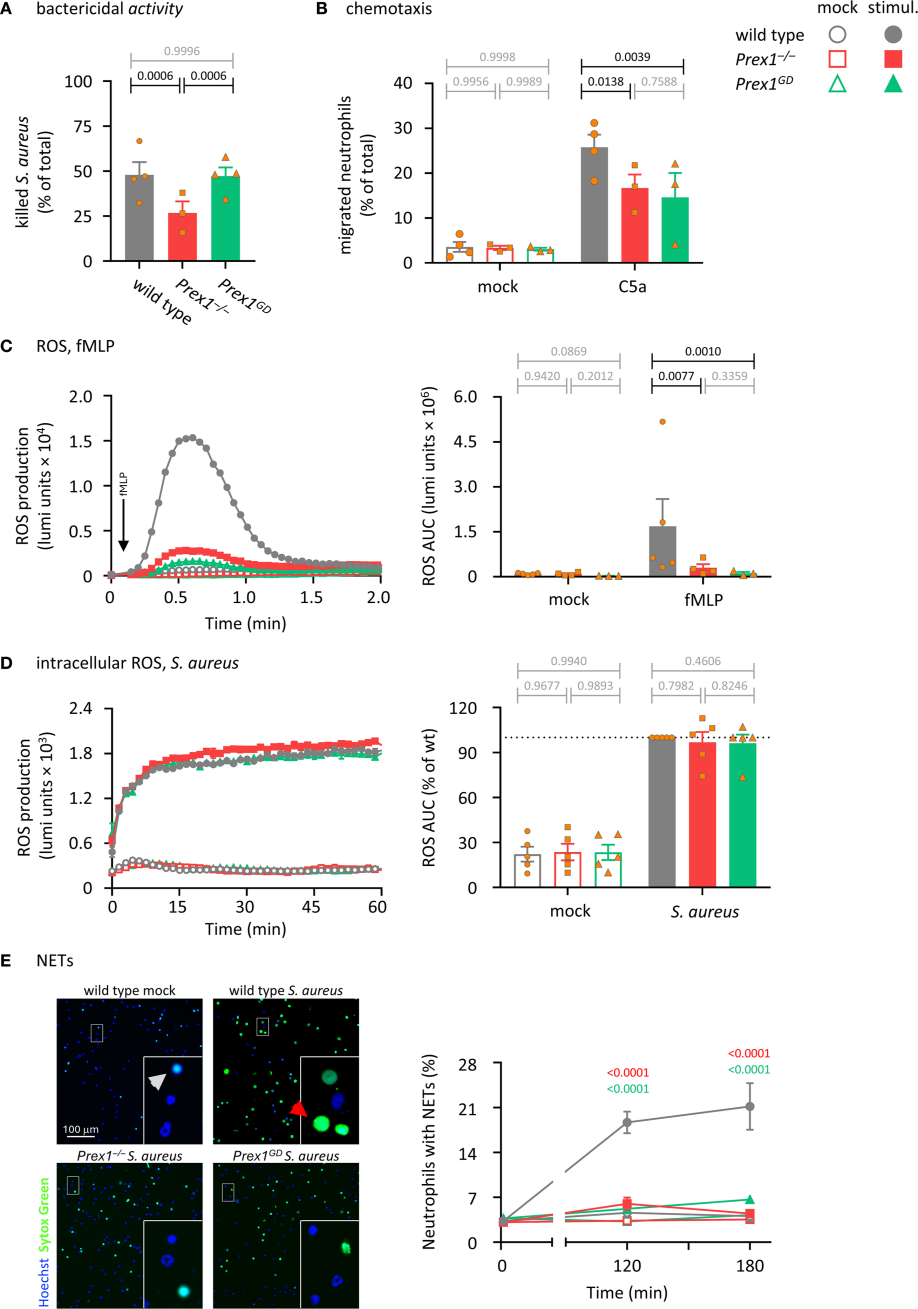
P-Rex1 mediates the killing of *S. aureus* by neutrophils independently of its catalytic Rac-GEF activity, whereas chemotaxis, ROS, and NETs require its Rac-GEF activity. **(A)** Bactericidal activity. Purified neutrophils from *Prex1* ^–/–^ (red squares), *Prex1* ^GD^ (green triangles), and wild type mice (grey circles) were primed with 20 ng/ml TNFα and 50 ng/ml GM-CSF for 45 min before incubation with serum-opsonized *S. aureus* for 90 min at a ratio of 1.5 bacteria per neutrophil. Heat-killed neutrophils were used as negative controls. Surviving bacteria were grown overnight and CFU enumerated. The % killing of bacteria by live neutrophils compared to heat-killed controls is plotted. Data are mean ± SEM of 3–4 independent experiments; each symbol represents the mean of one experiment. Statistics are one-way ANOVA with Tukey’s multiple comparisons test on log-transformed raw data; black p-values are significant, grey p-values non-significant. **(B)** Chemotaxis. Bone marrow cells from *Prex1* ^–/–^, *Prex1* ^GD^, and wild type mice were primed with 20 ng/ml TNFα and 50 ng/ml GM-CSF for 45 min before being stimulated with 3 nM C5a in transwell filters for 40 min, or mock stimulated. Transmigrated cells were analyzed by flow cytometry in parallel to control cells, using Ly6G^hi^/Mac1^hi^ staining to identify neutrophils. Data are mean ± SEM of 3–4 independent experiments; each symbol represents the mean of one experiment. Statistics are two-way ANOVA with Sidak’s multiple comparisons test on raw data. **(C)** fMLP-stimulated ROS production. Purified neutrophils as in **(A)** were primed with 1 μg/ml LPS for 90 min and then stimulated with 3 µM fMLP (filled symbols), or mock-stimulated (open symbols). ROS production was measured by real-time chemiluminescence assay with luminol and HRP for extra- and intracellular ROS. Left-hand panel shows luminometer traces from one representative experiment; right-hand panel shows the quantification as AUC over 2 min. Data are mean ± SEM of 3–5 independent experiments; each symbol represents the mean AUC from one experiment. Statistics are two-way ANOVA with Sidak’s multiple comparisons tests on log-transformed raw data. **(D)** *S. aureus*-stimulated intracellular ROS. Neutrophils were primed with 20 ng/ml TNFα and 50 ng/ml GM-CSF for 45 min in the presence of 50 units/ml SOD and 2000 units/ml catalase to scavenge extracellular ROS and were then stimulated with *S. aureus* at a ratio of 10 bacteria per neutrophil (filled symbols), or mock-stimulated (open symbols). ROS production was measured as in **(C)** except without HRP and in the presence of SOD and catalase, and quantification was done over 60 min. Data are mean ± SEM of 4 independent experiments; statistics are two-way ANOVA with Sidak’s multiple comparisons tests. **(E)** Formation of NETs. Neutrophils were seeded onto glass slides and allowed to adhere for 30 min before stimulation with serum-opsonised *S. aureus* at a ratio of 10 bacteria per neutrophil (closed symbols), or mock stimulation (open symbols). Non-cell permeable Sytox Green and cell-permeable Hoechst 33342 DNA dyes were added to samples 15 min before the end of the incubation, and cells were live-imaged by wide-field microscopy. Left-hand panel shows representative images from one experiment after 120 min stimulation or mock stimulation. Insets are magnifications of the indicated areas. Red arrows highlight NETs, white arrows dead cells without NETs. Right-hand panel shows quantification of NETs by ImageJ. Data are mean ± SEM of 3–4 independent experiments. Statistics are two-way ANOVA with Sidak’s multiple comparisons tests on raw data; significant p-values between and *Prex1* ^–/–^ and wild type are indicated in red, and between *Prex1* ^GD^ and wild type in green. For all panels, closed symbols show stimulated cells, open symbols mock-treated cells.

To investigate neutrophil responses, we began by testing the importance of P-Rex1 for the bactericidal activity of isolated neutrophils. Neutrophils from wild type*, Prex1* ^–/–^, and *Prex1* ^GD^ mice were primed with TNFα and GM-CSF, incubated with serum-opsonized *S. aureus*, and their ability to kill the bacteria was assessed by counting surviving bacterial CFU. The bactericidal activity of *Prex1* ^–/–^ neutrophils was reduced, whereas that of *Prex1* ^GD^ neutrophils was normal (**Figure 2A**). These data suggested that P-Rex1 mediates the killing of bacteria by isolated neutrophils independently of its catalytic Rac-GEF activity, as it does the clearance of *E. coli in vivo*.

To test if neutrophil chemotaxis depends on the Rac-GEF activity of P-Rex1, we performed transwell assays. Bone marrow cells from wild type, *Prex1* ^–/–^, *Prex1* ^GD^ mice were primed with TNFα and GM-CSF, plated into a transwell chamber, and stimulated with the chemoattractant C5a, an anaphylatoxin and ligand of the GPCR C5aR1. Cells that migrated through the 3 µm pores within 40 min were analyzed by flow cytometry. Both *Prex1* ^–/–^ and *Prex1* ^GD^ neutrophils had a reduced ability to migrate towards C5a (**Figure 2B**), which showed that P-Rex1 mediates neutrophil chemotaxis through its Rac-GEF activity.

Neutrophils kill bacteria through a combination of four effector responses, namely the production of ROS and NETs, degranulation and phagocytosis. We assessed these responses in turn, starting with ROS. P-Rex1 is known to mediate ROS production in response to the stimulation of GPCRs (1, 2, 15). Here, we tested ROS production stimulated with fMLP, a chemotactic peptide derived from bacteria such as *E. coli* or from the mitochondria of damaged eukaryotic cells, which signals through the GPCRs FPR1 and FPR2 (54). Purified neutrophils were primed with LPS and stimulated with fMLP, or mock-stimulated, while ROS production was measured by real-time chemiluminescence assay. The ability of both *Prex1* ^–/–^ and *Prex1* ^GD^ neutrophils to produce ROS in response to fMLP was strongly reduced (**Figure 2C**), which showed that P-Rex1 mediates GPCR-dependent neutrophil ROS formation through its Rac-GEF activity.

We previously reported that P-Rex1 is dispensable for ROS production in response to particle stimuli such as *S. aureus* bacteria, zymosan yeast particles, or sheep red blood cells, which required Rac-GEFs from the Vav family instead (16). Here we investigated further if P-Rex1 may be involved in intracellular ROS production specifically, as opposed to the total (extra- and intracellular combined) ROS production assessed previously. We primed neutrophils with TNFα and GM-CSF before adding catalase and superoxide dismutase (SOD) to scavenge extracellular ROS, stimulating the cells with serum-opsonized *S. aureus*, IgG-opsonized zymosan, or two different sizes of IgG-opsonized latex beads, and measuring intracellular ROS production over 60 min. P-Rex1 was dispensable for particle-induced intracellular ROS, regardless of whether the cells were stimulated with *S. aureus*, zymosan, or beads (**Figure 2D** and **Supplementary Figures 2A-C**). Neutrophils can also produce ROS independently of receptors, upon activation of protein kinase C with PMA. P-Rex1 is dispensable for this receptor-independent ROS response (2). We verified that PMA-stimulated ROS was also normal in *Prex1* ^GD^ neutrophils (**Supplementary Figure 2D**). Together with the particle-induced ROS data, this confirmed that the NADPH oxidase complex which produces ROS is intact in both *Prex1* ^–/–^ and *Prex1* ^GD^ neutrophils, and any defects are therefore due to roles of P-Rex1 in upstream signaling pathways. Consistent with the normal intracellular ROS production, particle-induced changes in cell shape appeared the same in all genotypes, when assessed by ImageStream analysis of neutrophils after the ROS assays (**Supplementary Figure 3**).

As P-Rex1 was required for the killing of *S. aureus* but not intracellular ROS production in response to *S. aureus*, we investigated if P-Rex1 controls the ROS-independent part of killing, by including the ROS inhibitor diphenyleneiodonium (DPI) in killing assays. As before, killing of *S. aureus* was reduced in *Prex1* ^–/–^ neutrophils, but DPI inhibited the killing in all genotypes equally, by 25% (interaction p-value 0.0895) (**Supplementary Figure 4**), confirming that P-Rex1 is required for the ROS-independent but not the ROS-dependent part of the killing response.

Next, we tested the formation of NETs. The role of P-Rex1 in this response has not been studied before. Purified neutrophils were incubated with serum-opsonized *S. aureus*, and NET formation was assessed by comparing signals from the non-cell permeable DNA dye Sytox Green and the cell-permeable DNA dye Hoechst 33342 using live-imaging. Both *Prex1* ^–/–^ and *Prex1* ^GD^ neutrophils had a reduced ability to form NETs (**Figure 2D**), which showed that P-Rex1 mediates NET formation and does so through its Rac-GEF activity. As the live-imaging only allowed us to monitor DNA release, but not the release of citrullinated histones and granule proteins associated with NETs, we additionally used a western blotting-based NETs assay (50). The *S. aureus*-induced release of the azurophil granule protein MPO was unaffected by P-Rex1, but the release of DNA and citrullinated histone 3 (CitH3) was reduced in both *Prex1* ^–/–^ and *Prex1* ^GD^ neutrophils (**Supplementary Figure 5**), confirming the results obtained by microscopy, although the phenotype here was not as stark. Therefore, P-Rex1 is required for NETs formation in a Rac-GEF activity-dependent manner.

To measure degranulation, we assessed the secretion of gelatinase (Mmp9) from gelatinase granules, lactoferrin from specific granules, and myeloperoxidase (MPO) from azurophil granules. Purified wild type and *Prex1* ^–/–^ neutrophils were stimulated with serum-opsonized *E. coli*, and proteins secreted into the cell supernatant were analyzed by Western blotting. P-Rex1 was dispensable for the secretion of gelatinase, lactoferrin, and MPO in response to serum-opsonized *E. coli* **(Supplementary Figure 6**), so does not control the degranulation of any type of neutrophil granule.

### P-Rex1 mediates the phagocytosis of IgG-opsonized zymosan yeast particles independently of its Rac-GEF activity

**Figure 3:**
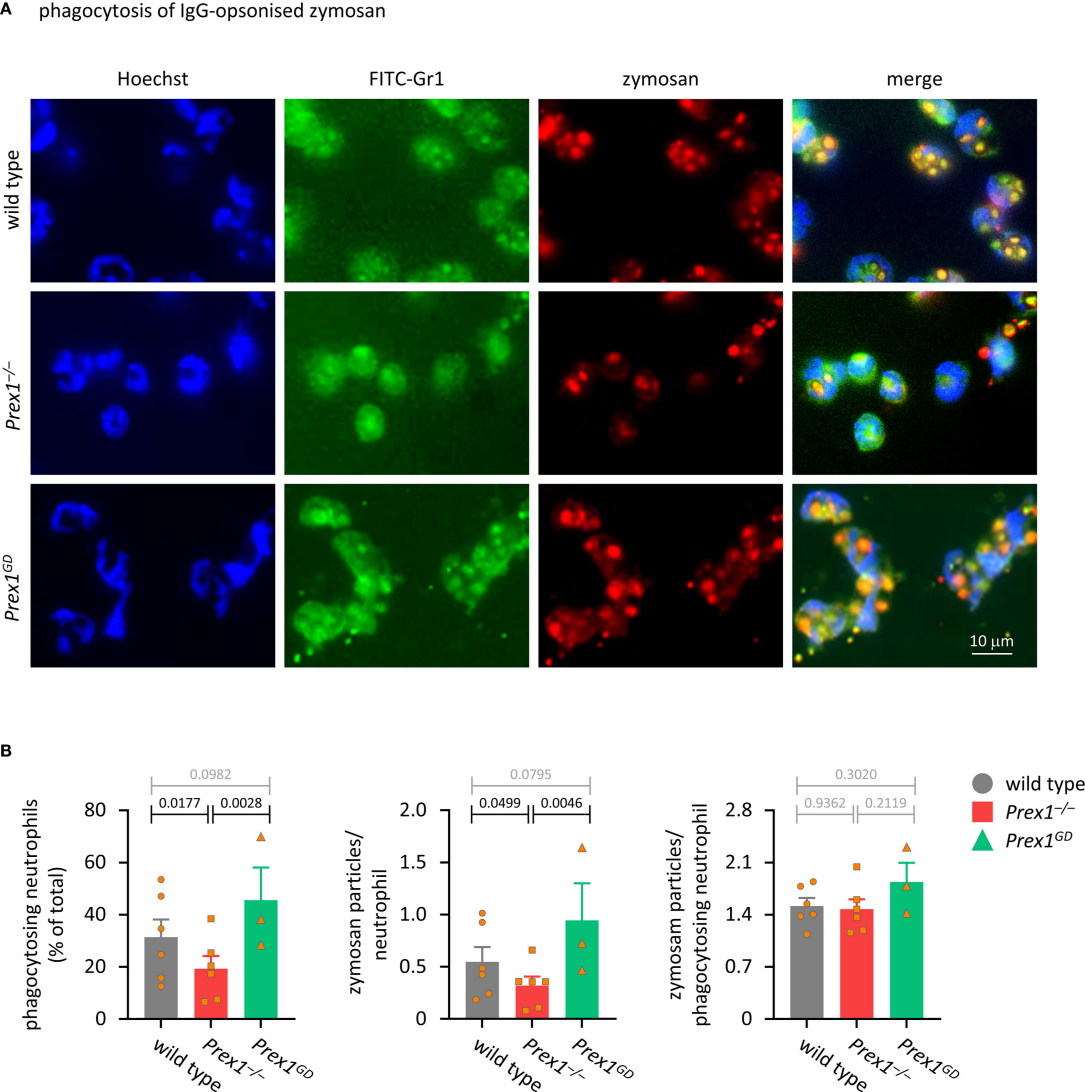
P-Rex1 mediates the phagocytosis of IgG-opsonised zymosan yeast particles independently of its Rac-GEF activity. **(A, B)** Phagocytosis of IgG-opsonised zymosan. Purified neutrophils from *Prex1* ^–/–^ (red squares), *Prex1* ^GD^ (green triangles), and wild type mice (grey circles) were allowed to adhere to glass coverslips for 15 min and stimulated with IgG-opsonized zymosan yeast particles for 30 min at a ratio of 5 particles per neutrophil. Cells were fixed and stained with FITC-Gr1 and AF568-IgG antibodies, as well as Hoechst 33342 DNA dye. Zymosan internalization was analyzed by wide-field microscopy and quantified using ImageJ. **(A)** Representative images from one experiment; **(B)** Quantification by ImageJ analysis for (left) percentage of neutrophils that phagocytosed at least one zymosan particle, (middle) mean number of phagocytosed particles in all neutrophils, (right) mean number of phagocytosed particles per phagocytosing neutrophils. Data are mean ± SEM of 3–6 independent experiments, each symbol represents the mean of one experiment. Statistics are one-way ANOVA with Tukey’s multiple comparisons tests on log-transformed raw data; black p-values are significant, grey p-values non-significant.

A role for P-Rex1 in phagocytosis has not been reported. Here, we tested phagocytosis in various ways, starting with the phagocytosis of IgG-opsonized zymosan yeast particles, which are taken up upon activation of Fc receptors by the IgG opsonin, as well as through other types of receptors (55). The ability of *Prex1* ^–/–^ neutrophils to phagocytose IgG-opsonized zymosan was reduced, whereas phagocytosis was normal in *Prex1* ^GD^ neutrophils (**Figures 3A, B**). Therefore, P-Rex1 is required for phagocytosis, and it mediates this response independently of its catalytic Rac-GEF activity. This was seen both when we analyzed the percentage of neutrophils that took up at least one particle and the number of zymosan particles taken up per neutrophil (**Figure 3B**). In contrast, the number of particles inside phagocytosis-competent neutrophils was normal, which suggested that P-Rex1 controls mostly the likelihood of the phagocytosis response occurring.

### P-Rex1 mediates the Fc receptor-dependent and integrin-dependent phagocytosis of IgG-opsonized latex beads

**Figure 4:**
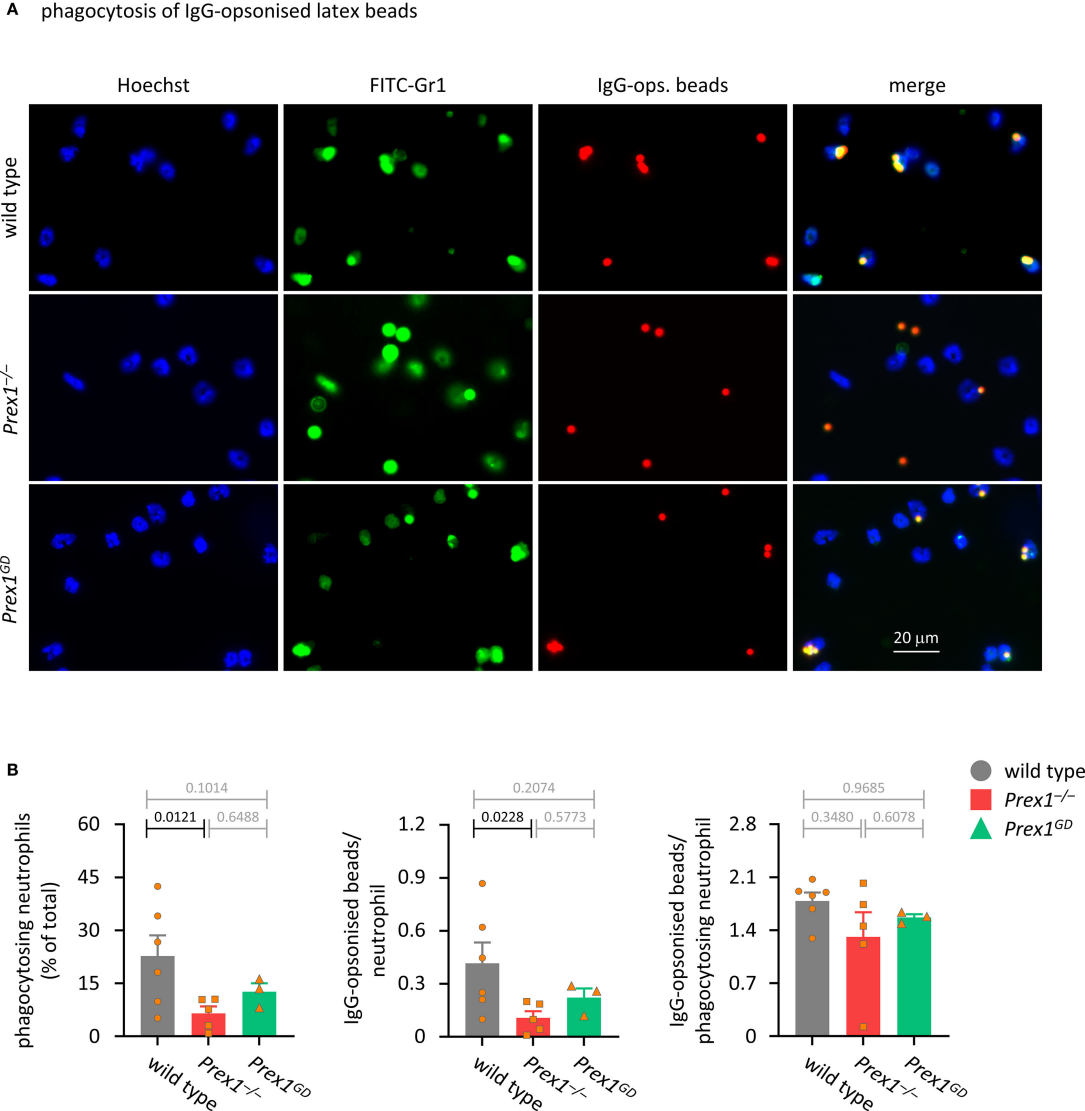
P-Rex1 mediates the phagocytosis of IgG-opsonised latex beads in part independently of its Rac-GEF activity. **(A, B)** Phagocytosis of IgG-opsonised latex beads. Purified neutrophils from *Prex1* ^–/–^ (red squares), *Prex1* ^GD^ (green triangles), and wild type mice (grey circles) were primed with 20 ng/ml TNFα and 50 ng/ml GM-CSF for 45 min, allowed to adhere to glass coverslips for 15 min, and stimulated with IgG-opsonized latex beads at a ratio of 10 particles per neutrophil for 60 min before fixing, staining, imaging, and image analysis as in **Figure 3**. Data are mean ± SEM of 3–6 independent experiments, each symbol represents the mean of one experiment. Statistics are one-way ANOVA with Tukey’s multiple comparisons tests on log-transformed raw data; black p-values are significant, grey p-values non-significant.

Next, we tested the phagocytosis of latex beads, which are relatively inert particles until opsonized, making it easier to delineate the receptor types involved. First, we used IgG-opsonized latex beads, which are largely phagocytosed in response to the activation of Fc receptors. Unsurprisingly, phagocytosis of the latex beads was less efficient than that of zymosan, and neutrophils needed to be primed to phagocytose these particles (**Supplementary Figure 7**). As with zymosan, the ability of *Prex1* ^–/–^ neutrophils to phagocytose IgG-opsonized latex beads was reduced, which confirmed that P-Rex1 is required for phagocytosis of different types of particles (**Figures 4A, B**). Again, both the percentage of neutrophils that took up at least one bead, and the number of beads per neutrophil were reduced in neutrophils, whereas the number of beads per phagocytosis-competent neutrophil was normal (**Figure 4B**). Phagocytosis of IgG-opsonized latex beads also appeared somewhat reduced in *Prex1* ^GD^ neutrophils, but this reduction was smaller than in *Prex1* ^–/–^ cells and not significant, neither when compared to *Prex1* ^+/+^ nor *Prex1* ^–/–^ (**Figure 4B**). Therefore, P-Rex1 mediates the phagocytosis of IgG-opsonized latex beads, and it appears to do so at least in part independently of its catalytic Rac-GEF activity.

**Figure 5:**
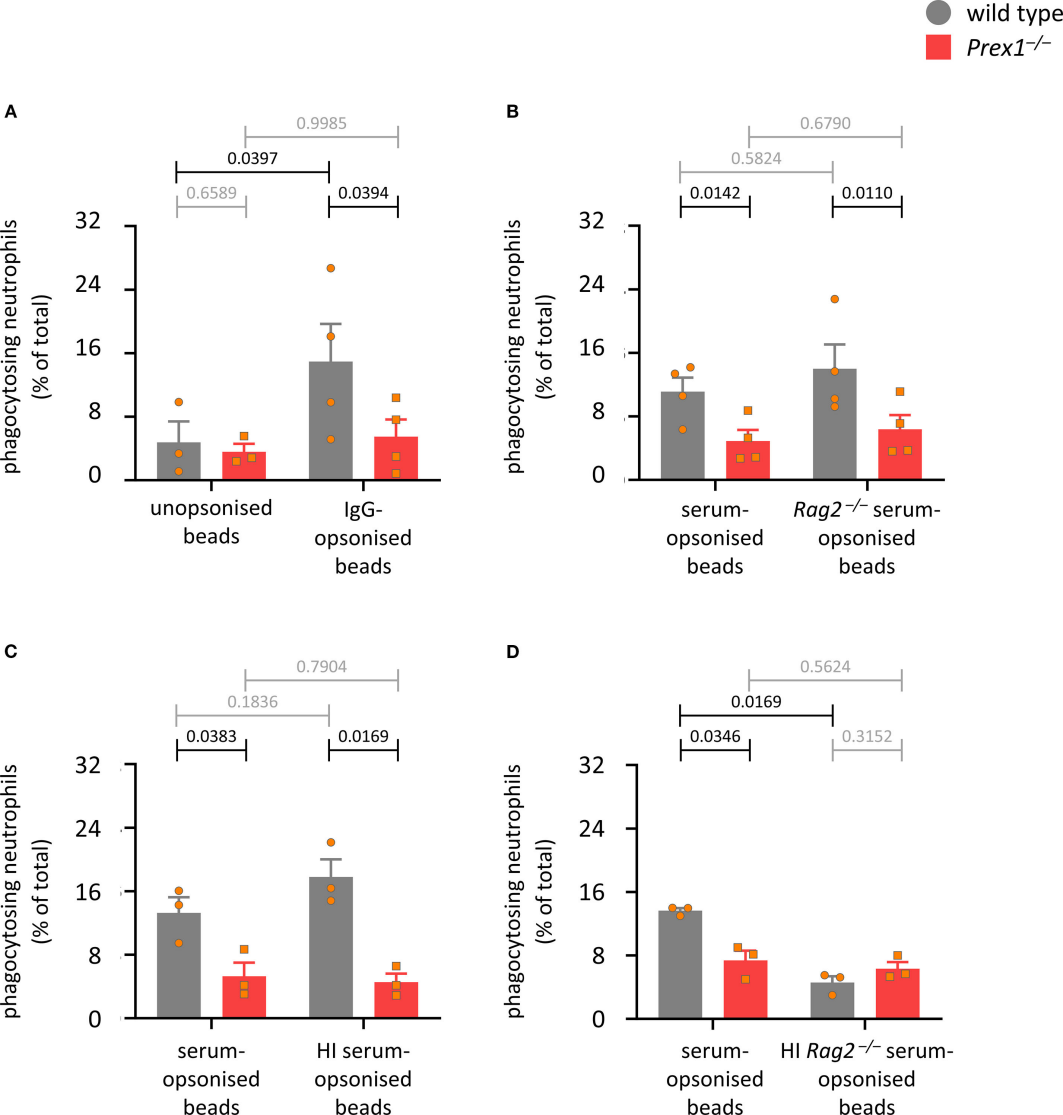
P-Rex1 mediates the integrin- and Fc receptor-dependent phagocytosis of latex beads. **(A-D)** Phagocytosis of latex beads. Purified neutrophils from *Prex1* ^–/–^ (red squares) and wild type mice (grey circles) were primed with 20 ng/ml TNFα and 50 ng/ml GM-CSF for 45 min, allowed to adhere to glass coverslips for 15 min, and stimulated with latex beads that had or had not been opsonized with various reagents, before phagocytosis was measured as in **Figure 4** **(A)** comparing unopsonized and IgG-opsonized latex beads; **(B)** comparing opsonization with serum from wild type and *Rag2^–/–^* mice; **(C)** comparing opsonization with serum from wild type mice with and without prior heat-inactivation of complement factors; **(D)** comparing opsonization with serum from *Rag2^–/–^* mice with and without prior heat-inactivation of complement factors. Data are mean ± SEM of 3–4 independent experiments for each panel; each symbol represents the mean of one experiment. Statistics are two-way ANOVA with Sidak’s multiple comparisons tests on square root-transformed raw data; black p-values are significant, grey p-values non-significant.

Phagocytosis occurs mainly in response to the activation of two types of receptors, Fc receptors and integrins. To investigate which phagocytosis pathway depends on P-Rex1, we assessed the ability of *Prex1* ^–/–^ neutrophils to phagocytose latex beads opsonized by a range of different methods. First, we compared unopsonized and IgG-opsonized beads. Uptake of unopsonized beads was very inefficient, and the beads had to be opsonized to reveal a role for P-Rex1. This was seen both when assessing the percentage of phagocytosing neutrophils (**Figure 5A**) and the number of beads per neutrophil (**Supplementary Figure 8A**). To assess the role of P-Rex1 in integrin-dependent phagocytosis, we compared beads opsonized with mouse serum, which activates both Fc receptors and integrins, to beads opsonized with serum from *Rag2^–/–^* mice, which is deficient in immunoglobulins and so should only stimulate integrin-dependent phagocytosis. P-Rex1 was required for phagocytosis under both conditions (**Figure 5B** and **Supplementary Figure 8B**), suggesting that it mediates integrin-dependent phagocytosis. To assess the role of P-Rex1 in Fc receptor dependent phagocytosis, we compared beads opsonized with mouse serum to beads opsonized with mouse serum that had been heat-inactivated to eliminate the complement factors that activate neutrophil integrins. Again, P-Rex1 was required for phagocytosis under both conditions (**Figure 5C** and **Supplementary Figure 8C**), suggesting that P-Rex1 mediates Fc receptor-dependent phagocytosis. To see if P-Rex1 mediates other potential phagocytosis pathways than the integrin and Fc receptor pathways, we compared beads opsonized with mouse serum to beads opsonized with *Rag2^–/–^* mouse serum that had been heat-inactivated to eliminate both immunoglobulins and complement factors. Under the latter condition, phagocytosis was very inefficient, as expected, and a role for P-Rex1 was no longer observed (**Figure 5D** and **Supplementary Figure 8D**). Hence, P-Rex1 is required for both Fc receptor- and integrin-dependent phagocytosis in neutrophils.

### P-Rex1 does not regulate the cell surface levels of phagocytosis receptors Mac1, FcγRIII, and FcγRII, nor the agonist-induced internalization of CXCR4 and C5aR1

Major phagocytosis receptors on the neutrophil surface are the β2 integrin Mac1 (CD11b/CD18) and the Fc receptors FcγRIII (CD16) and FcγRII (CD32) (55–58). We previously used flow cytometry to show that P-Rex1 does not control the cell surface level of Mac1 (16). Here, we confirmed this under unprimed, mock-primed and primed conditions. Priming with TNFα and GM-CSF led to the upregulation of Mac1 as expected, but the surface level of Mac1 was the same between wild type and *Prex1* ^–/–^ neutrophils under all conditions (**Supplementary Figure 9A**). For FcγRIII and FcγRII, mock priming (incubation at 37 ˚C) led to a small reduction in their surface levels, whereas priming did not affect them further, but again the levels of these receptors were the same between wild type and *Prex1* ^–/–^ neutrophils under all conditions (**Supplementary Figures 9B, C**). Therefore, P-Rex1 does not control phagocytosis by regulating the levels of the principal phagocytosis receptors on the neutrophil surface.

We recently showed that P-Rex1 limits the agonist-induced internalization of GPCRs independently of its catalytic Rac-GEF activity (48), which is relevant here as GPCRs regulate the activity of integrins. To investigate if P-Rex1 controls the trafficking of the GPCRs CXCR4 and C5aR1 in neutrophils, we stimulated their internalization with SDF1α and C5a, respectively. Upon SDF1α stimulation, CXCR4 was internalized to the same extent in *Prex1* ^–/–^ and wild type neutrophils, except at the 20 min timepoint, where *Prex1* ^–/–^ cells showed greater internalization (**Supplementary Figure 10A**). No significant role for P-Rex1 was seen in the C5a-stimulated internalization of C5aR1. However, C5aR1 is one of the fastest GPCR to be internalized (51), being maximally internalized within 2 min of agonist-stimulation, and we could see a trend for increased internalization at that timepoint when we fixed cells prior to analysis to prevent any further trafficking (**Supplementary Figure 10B**). Overall, any role of P-Rex1 in the trafficking of these two neutrophil GPCRs at least seems too minor to underlie its function in integrin- and Fc receptor- dependent phagocytosis. Of note, we also recently showed that P-Rex1 plays a Rac-GEF independent role in glucose uptake in hepatocytes (5). However, glucose uptake was normal in *Prex1* ^–/–^ neutrophils under the conditions tested (**Supplementary Figure 11**), suggesting that this role is also unrelated to the function of P-Rex1 in phagocytosis.

### P-Rex1 is required for the optimal activation of Syk, but not Erk or p38 Mapk, by IgG-opsonized latex beads

**Figure 6:**
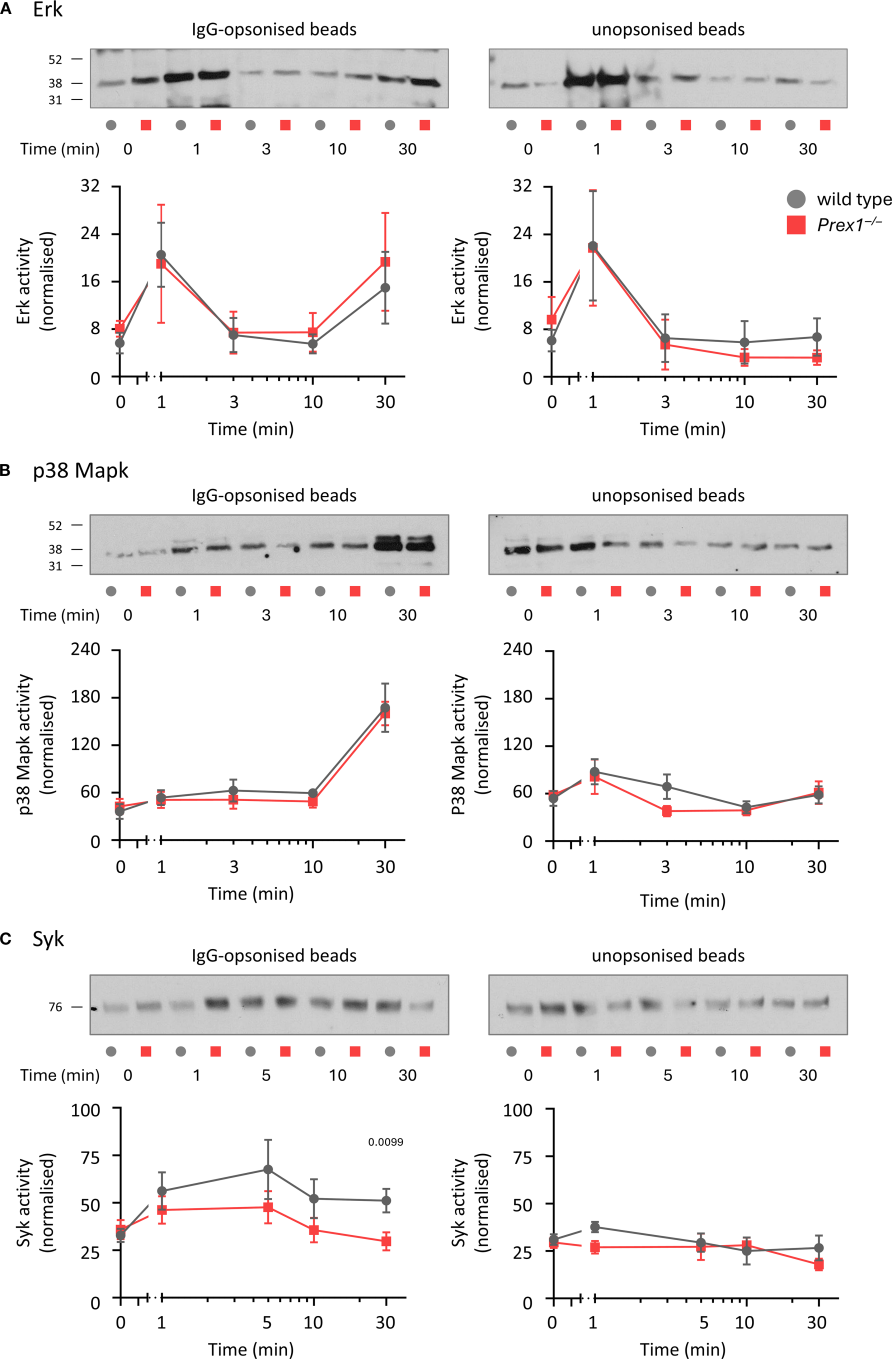
P-Rex1 is required for the optimal activation of Syk, but not Erk or p38 Mapk, by IgG-opsonized latex beads. **(A-C)** Signalling pathways. Purified neutrophils from *Prex1* ^–/–^ (red squares) and wild type mice (grey circles) were primed with 20 ng/ml TNFα and 50 ng/ml GM-CSF for 45 min before incubation for 30 min in the presence of IgG-opsonized (left) or unopsonized (right) beads for the indicated periods of time towards the end of the incubation. Total cell lysates were analyzed by Western blotting for **(A)** phospho-Erk1/2, **(B)** phospho-p38 Mapk and **(C)** phospho-Syk. The activities of Erk, p38 Mapk and Syk were quantified using Fiji densitometry by dividing the phospho-signals for each protein by the protein loading (coomassie) over the whole lane. Representative blots comparing IgG-opsonized and unopsonized beads from the same experiment are shown. Data are mean ± SEM of 5 independent experiments for **(A)**, 4 for **(B)**, and 6 for **(C)**. Statistics are two-way ANOVA with Sidak’s multiple comparisons tests on log-transformed raw data; significant p-values are indicated.

As P-Rex1 controls both Fc receptor-dependent and integrin-dependent phagocytosis, we investigated common signaling pathways downstream of these receptors, which include Erk, p38 Mapk, and Syk. We tested these pathways by Western blotting, comparing responses between IgG-opsonized and unopsonized latex beads. Erk was activated by both IgG-opsonized and unopsonized beads, although only IgG-opsonized beads elicited a second wave of activation after 30 min, whereas p38 Mapk and Syk were activated by IgG-opsonized beads alone. P-Rex1 had no influence on the Erk and p38 Mapk pathways, but the activation of Syk by IgG-opsonized beads was lower in *Prex1* ^–/–^ cells than in wild type cells (**Figure 6**). Therefore, P-Rex1 is required for the activation of Syk by immunoglobulin-opsonized particles. To test furthermore whether this requires the Rac-GEF activity of P-Rex1, we compared Syk activity stimulated with IgG-opsonized beads in wild type, *Prex1* ^–/–^, and *Prex1* ^GD^ neutrophils. Both *Prex1* ^–/–^ and *Prex1* ^GD^ neutrophils showed reduced activation of Syk by IgG-opsonized latex beads (**Supplementary Figure 12**), which revealed that P-Rex1 controls Syk through its Rac-GEF activity.

### P-Rex1 mediates the activation of Rac by fMLP or IgG-opsonized latex beads through its Rac-GEF activity

**Figure 7:**
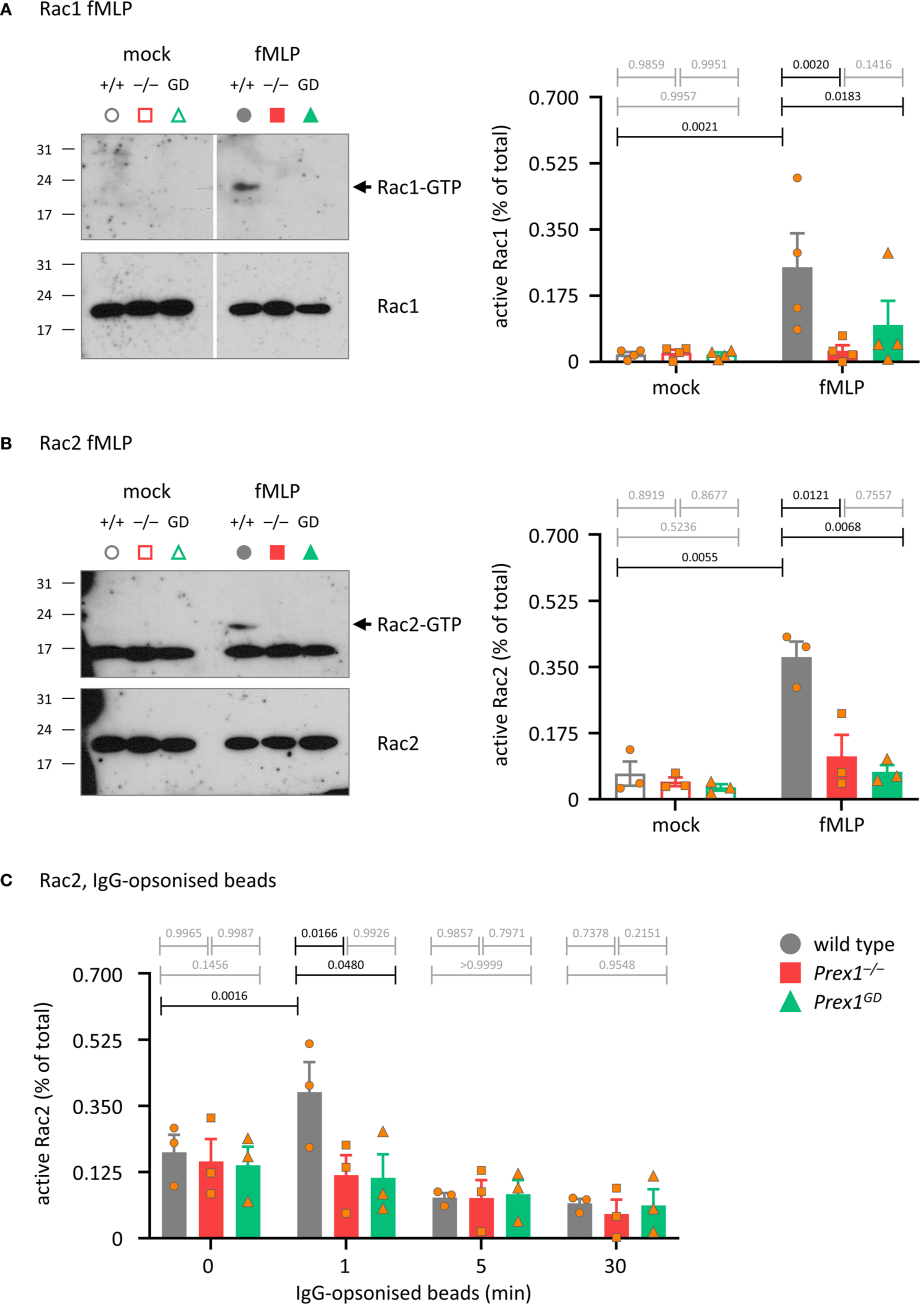
P-Rex1 mediates the activation of Rac by fMLP and IgG-opsonized latex beads through its Rac-GEF activity. **(A, B)** fMLP. Purified neutrophils from *Prex1* ^–/–^ (red squares), *Prex1* ^GD^ (green triangles), and wild type mice (grey circles) were stimulated with 10 μM fMLP for 10 s **(A)**, or 5 s **(B)** at 37°C (closed symbols), or mock-stimulated (open symbols). Total lysates were prepared and active, GTP-bound, active Rac1 **(A)** and Rac2 **(B)** were isolated from total lysates by Pak-CRIB pull down and quantified by Western blotting with Rac1 and Rac2 antibodies and Fiji densitometry. 1.25% of the total lysate were loaded as control. The GTP-Rac and total Rac membranes for each protein were processed together in the same containers throughout to allow direct comparison. The GTP-Rac signal was normalized to the total Rac signal for each lane. Representative blots from one experiment are shown. Data are mean ± SEM of 4 independent experiments in **(A)** and 3 in **(B)**; each symbol represents one experiment. **(C)** IgG-opsonized latex beads. Neutrophils were primed with 20 ng/ml TNFα and 50 ng/ml GM-CSF for 45 min prior to incubation with IgG-opsonized beads for the indicated periods of time at a ratio of 10 beads per neutrophil. Rac2 activity was analyzed as in **(A, B)**. Data are mean ± SEM of 3 independent experiments; each symbol represents one experiment. Statistics in **(A-C)** are two-way ANOVA with Sidak’s multiple comparisons tests on square root-transformed raw data; black p-values are significant, grey p-values non-significant.

Rac is required for both Fc receptor-dependent and integrin-dependent phagocytosis (59), Therefore, we tested the role of P-Rex1 in Rac activation, comparing *Prex1* ^–/–^ and *Prex1* ^GD^ neutrophils. First, we investigated GPCR-dependent activation of Rac, as P-Rex1 is known to mediate Rac1 and Rac2 activities in this pathway. As expected, the fMLP-stimulated activation (GTP-loading) of both Rac1 and Rac2 was reduced in *Prex1* ^–/–^ and Prex1^GD^ neutrophils, confirming that the catalytic Rac-GEF activity of P-Rex1 mediates GPCR-dependent Rac signaling (**Figures 7A, B**). Finally, we tested Rac activation in response to IgG-opsonized latex beads. For technical reasons (IgG interfering with Rac1 blots), we could only investigate Rac2 in this context. In wild type neutrophils, Rac2 was activated by IgG-opsonized beads within 1 min, whereas no activation was seen in *Prex1* ^–/–^ or Prex1^GD^ neutrophils (**Figure 7C**). Therefore, P-Rex1 is required for the activation of Rac2 in response to immunoglobulin-opsonized particles and mediates this response through its Rac-GEF activity. These data implied furthermore that the GEF-activity independent role of P-Rex1 in phagocytosis is unlikely to involve Rac2.

## Discussion

Our study identified new roles for P-Rex1 in neutrophil phagocytosis and NET formation. It showed that P-Rex1 mediates chemotaxis, ROS production and NET formation through its catalytic Rac-GEF activity, but it controls the clearance of *E. coli* during septic peritonitis, as well as neutrophil bactericidal activity and phagocytosis, independently of its catalytic Rac-GEF activity.

*Prex1* ^–/–^ mice showed impaired clearance of pathogenic *E. coli* during acute peritoneal infection despite increased neutrophil recruitment. We previously reported normal pulmonary neutrophil recruitment in *Prex1* ^–/–^ mice infected with *S pneumoniae* but reduced recruitment into LPS-inflamed lungs when Vav1 was co-deleted, and during peritonitis induced by thioglycollate (2, 20, 60). Therefore, the requirement for P-Rex1 in neutrophil recruitment varies depending on the organ environment and inflammatory stimulus. The increased recruitment observed here during *E. coli* peritonitis likely reflects an increased inflammatory state caused by the higher bacterial load in *Prex1* ^–/–^ mice. As neutrophil recruitment was not reduced, it seems likely that impaired neutrophil effector functions contributed to the poor immunity of *Prex1* ^–/–^ mice. However, it should be noted that neutrophils are not the only cells conferring antibacterial immunity in this acute peritonitis model, resident alveolar macrophages also contribute, so potential GEF-activity independent roles of P-Rex1 in these cells should also be evaluated in the future. It would be valuable to generate mice with cell-lineage-specific deletion or catalytic inactivity of P-Rex1 to address the relative contributions of P-Rex1 and its catalytic activity in neutrophils, macrophages, platelets, and endothelial cells to infection and inflammation *in vivo.*

Neutrophil chemotaxis was reduced in *Prex1* ^–/–^ neutrophils in a Rac-GEF activity dependent manner, although neutrophil recruitment *in vivo* was enhanced. Chemotaxis and recruitment cannot be compared directly, as chemotaxis is only one of numerous, complex adhesion and migration processes required for neutrophil recruitment to sites of inflammation. Chemotaxis mainly plays a role late in the recruitment cascade, after the extravasation of neutrophils from the blood stream, during their active migration within the inflamed or infected target tissues (61). In the peritoneum, the peritoneal wall is the only layer of tissue between blood vessels and peritoneal cavity lumen, so chemotaxis plays less of a role.

The impairment in immunity against *E. coli* infection was seen in *Prex1* ^–/–^ but not *Prex1^GD^* mice, so was independent of the Rac-GEF activity. The killing of *E. coli* by neutrophils occurs through both ROS-dependent and ROS-independent mechanisms, depending on the *E. coli* strain, intra- versus extracellular killing, and *in vitro* or *in vivo* conditions (62–65). It remains to be seen through which mechanism P-Rex1 deficiency impacts the clearance of *E. coli in vivo*. The killing of *S. aureus* by isolated neutrophils, which also occurs through both ROS-dependent and ROS-independent mechanisms (66), was impaired in *Prex1* ^–/–^ but not *Prex1^GD^* neutrophils. Use of the ROS inhibitor DPI in killing assays showed that P-Rex1 is required for the ROS-independent part of the killing response, as DPI inhibited killing in all genotypes, including P*rex1* ^–/–^ neutrophils, to the same degree. As the killing of *S. aureus* was impaired in *Prex1* ^–/–^ but not *Prex1^GD^* neutrophils., we searched for P-Rex1-dependent neutrophil effector responses that did not require the Rac-GEF activity.

We identified a role for P-Rex1 in phagocytosis which was independent of the Rac-GEF activity. The percentage of phagocytosing *Prex1* ^–/–^ neutrophils was reduced, whereas the number of particles eaten by phagocytosis-competent neutrophils was normal, suggesting that P-Rex1 controls the likelihood of phagocytosis occurring rather than the efficacy of phagocytosis. This is reminiscent of the control of neutrophil chemotaxis by PI3Kγ, where the main role of PI3Kγ is to control the likelihood of neutrophils migrating rather than their speed or directionality (67). This role of PI3Kγ correlates with the ability of neutrophils to adhere and undergo F-actin polarization. We suspect that differences in F-actin structure also underlie the phagocytosis impairment in *Prex1* ^–/–^ neutrophils, as we previously identified defects in fMLP-induced F-actin polymerization and spreading in *Prex1* ^–/–^ neutrophils (2, 20). High-resolution imaging of the interactions between particle and neutrophil surface and of the forming phagosome will be required to increase our understanding of the role of P-Rex1 in this response in the future.

P-Rex1 was required for the phagocytosis of opsonized zymosan and latex beads. We used various opsonization methods to learn which phagocytosis receptors are involved. Opsonization with *Rag2^–/–^* serum, which lacks immunoglobulins, stimulates complement-dependent phagocytosis, with the main complement receptor in neutrophils being the β2 integrin Mac1, whereas opsonization with heat-inactivated serum, which lacks complement factors, stimulates Fc receptor-dependent phagocytosis. (55–58). P-Rex1 was required for both integrin- and Fc receptor-dependent phagocytosis. Fc receptors and integrins are the main but not the only phagocytosis receptors. Non-opsonic phagocytosis receptors, such as C-type lectin receptors, also contribute (57). In our study, phagocytosis of non-opsonized particles was very inefficient, but we saw no role for P-Rex1 under these conditions, so it appears that P-Rex1 mediates the Fc receptor-dependent and integrin-dependent response, but not opsonin-independent phagocytosis.

The cytoskeletal dynamics that underlie integrin- and Fc receptor-dependent phagocytosis differ. Generally, during integrin-dependent phagocytosis, particles sink into the cell, whereas Fc receptor activation causes the extension of pseudopodia outwards to encircle the particle (56). P-Rex1 is likely to govern underlying cytoskeletal processes common to both. The P-Rex1 dependent phagocytosis of IgG-opsonized zymosan was independent of the Rac-GEF activity, whereas this independence was less clear during the phagocytosis of IgG-opsonized latex beads. Differences between these particle types include size, the beads we used were 2 μm in diameter whereas zymosan particles vary in size but are generally larger and therefore require different cytoskeletal dynamics. Moreover, zymosan bears additional epitopes recognized by various receptor types, including integrins and non-phagocytic receptors that aid in phagocytosis, so P-Rex1 signaling downstream of these receptors may require the Rac-GEF activity less than Fc receptor signaling.

P-Rex1 is not the only neutrophil Rac-GEF important in phagocytosis. Vav3 mediates the phagocytosis of IgG-opsonized red blood cells (34), and Vav1 and Vav3 together mediate the phagocytosis of serum-opsonized *E. coli* (68). We recently showed that Dock2 is required for the phagocytosis of IgG-opsonized latex beads, controlling the percentage of phagocytosing cells, speed of engulfment, and number of particles taken up, and we used FRET imaging to show that the speed of engulfment correlates with the amount of Rac activity generated by Dock2 around the phagosome (26). It remains to be seen if these other Rac-GEFs play catalysis-dependent or -independent roles in phagocytosis. GEF activity-independent functions in other cell responses have been described for several GEFs, including the activation of NFAT by Vav1 in lymphocytes (69), control of dendrite morphology by Tiam1 (70), and regulation of cytokine production by Dock GEFs (71).

In addition to phagocytosis, we also investigated the other neutrophil effector responses. GPCR-dependent ROS production required the Rac-GEF activity of P-Rex1, which was expected as P-Rex1 activates Rac2, an integral component of the NADPH oxidase complex, in this pathway. We previously established that P-Rex1 is dispensable for total ROS production (extracellular and intracellular combined) stimulated by particles (16), and we confirmed here that this also holds true for intracellular ROS production specifically. Intracellular ROS is produced inside the maturing phagosome, after the NADPH oxidase assembles on the phagosome membrane, and is one of the mechanisms by which neutrophils kill bacteria following phagocytosis (56, 57). It is unclear why intracellular ROS, and the cell shape changes induced by particles during the ROS assays, were normal despite reduced phagocytosis. Perhaps phagocytosis-competent *Prex1* ^–/–^ cells compensated for reduced ROS production in others, although it is difficult to imagine how such compensation would occur. Importantly, bacteria-induced ROS production was normal, so it could not explain the Rac-GEF activity independent impairment in bactericidal activity of *Prex1* ^–/–^ neutrophils or the reduced innate immunity of *Prex1* ^–/–^ mice to *E. coli* infection.

ROS production is generally important for the formation of NETs. Considering that *S. aureus*-induced ROS production was normal, it was therefore surprising that *S. aureus* induced NETs were impaired in *Prex1* ^–/–^ and *Prex1^GD^* neutrophils, both by live-imaging and western blotting, suggesting that P-Rex1 mediates NETs through its Rac-GEF activity but independently of ROS. The NETs we observed by live-microscopy were cloud-like rather than filamentous. Neutrophils can make both types of NETs depending on handling and type of stimulus, and cloud-like NETs are typically seen by live imaging (72). NET formation can occur in response to numerous physiological stimuli, through peptidyl arginine deiminase 4, which catalyses histone citrullination to decondense chromatin (73, 74). The pathways through which P-Rex1 controls NETs require further investigation in the future. Importantly, as NET formation required the Rac-GEF activity of P-Rex1, it alone could not explain the impaired bactericidal activity of *Prex1* ^–/–^ neutrophils which was independent of the Rac-GEF activity.

P-Rex1 was dispensable for the degranulation of azurophil, specific and gelatinase granules induced by *E. coli*. Rac2 is required for the degranulation of azurophil granules (32), so another Rac-GEF, which remains to be identified, must activate Rac2 during this process. It is important to note that we only measured degranulation into the extracellular space, not into the phagosome. If P-Rex1 controls degranulation into the phagosome, then this could contribute to the reduced bactericidal activity in *Prex1* ^–/–^ neutrophils.

Phagocytosis was the only neutrophil effector response controlled by P-Rex1 independently of its catalytic Rac-GEF activity. This suggested that P-Rex1 mediates the bactericidal activity of neutrophils, which was also catalysis-independent, mainly through phagocytosis. However, phagocytosis alone does not kill bacteria. In other cell types, such as macrophages, the lumen of phagosomes acidifies during phagosome maturation, with antibacterial effects. However, the same acidification does not occur in neutrophils, because the typical lysosomes required to form phagolysosomes are largely replaced by the various granule types, and because phagosomal ROS production counteracts acidification (56, 75). In neutrophils, the proteases and other antibacterial proteins delivered into the phagosome by degranulation kill the pathogens, together with the ROS generated in the phagosome. *In vivo*, neutrophils can kill pathogens also indirectly by secreting cytokines and other inflammatory mediators to attract other types of immune cell, but this is not relevant in isolated neutrophils. As bacteria-induced ROS production, shape changes, and degranulation into the extracellular space were normal in *Prex1* ^–/–^ neutrophils, a likely scenario is that the reduced NET production in *Prex1* ^–/–^ and *Prex1^GD^* neutrophils, and the reduced phagocytosis in *Prex1* ^–/–^, combine to result in the impaired killing of bacteria remaining in the extracellular space around *Prex1* ^–/–^ neutrophils.

The Rac-GEF activity independent mechanisms through which P-Rex1 control phagocytosis remain unclear, but as P-Rex1 was required for both integrin- and Fc receptor-dependent phagocytosis, we investigated signaling pathways common to integrins and Fc receptors. The surface levels of the major relevant receptors, Mac1, FcγRIII, and FcγRII, were normal in *Prex1* ^–/–^ neutrophils. However, integrins like Mac1 adopt different conformations ranging from bent inactive to open active, their activation requiring inside-out signaling. We previously showed that P-Rex1 is required for the IL8-dependent activation of Mac1 in HL60 cells (18). However, β2-integrin activity is more difficult to assess in mouse neutrophils, as conformation-specific antibodies do not exist. One could assess ICAM1-binding and integrin clustering as proxies to investigate if P-Rex1 controls Mac1 activation independently of its Rac-GEF activity. It is conceivable that such effects on Mac1 would also impact Fc receptor-dependent phagocytosis, as there is crosstalk between these receptor types (57). We recently showed that P-Rex1 controls GPCR trafficking, limiting the agonist-induced internalization of these receptors independently of its Rac-GEF activity, in addition to mediating GPCR signaling through its Rac-GEF activity (48). This role in GPCR trafficking was seen with all GPCR types tested and in a range of cell types. As GPCRs mediate the activation of integrins, we investigated here the trafficking of two relevant neutrophil GPCRs, CXCR4 and C5aR1. However, the C5a-stimulated internalization of C5aR1 was normal, and effects of P-Rex1 on the SDF1-induced internalization of CXCR4 were small and only observed at a late stage of receptor trafficking. Unlike in other cell types, neutrophil degranulation delivers GPCRs to the plasma membrane at the same time as active GPCRs are being internalized, thus presumably masking the role of P-Rex1 in GPCR internalization. In any event, it seems unlikely that the small net effects of P-Rex1 on GPCR trafficking can explain the GEF-activity independent defects in phagocytosis and bactericidal activity.

Neutrophils use primarily glycolysis, rather than mitochondrial respiration, to produce the ATP that fuels chemotaxis, phagocytosis, degranulation, and the production of ROS and NETs. Additionally, chemotaxis and NETs are aided by mitochondrially-derived ATP, the pentose phosphate pathway makes the NADPH which is required for ROS and ROS-dependent NETs, and gluconeogenesis and glycogenesis generate intracellular glycogen stores required for effective survival and bacterial killing (76–78). We recently identified a Rac-GEF activity-independent role of P-Rex1 in hepatic glucose uptake, trafficking of the glucose transporter Glut2 and the glucose metabolism-regulating GPCR Gpr21, and mitochondrial metabolism of glucose to ATP (5). In contrast, we did not see a defect in glucose uptake in *Prex1* ^–/–^ neutrophils, presumably because other types of glucose transporter regulate glucose uptake in neutrophils. It would be interesting to evaluate metabolic roles of P-Rex1 in neutrophils in more detail in the future.

Syk is required for both Fc receptor- and integrin-dependent phagocytosis, and we identified Syk as a target of P-Rex1 during stimulation with IgG-opsonized latex beads, whereas P-Rex1 was dispensable for p38 Mapk and Erk activities. Research in monocytes and macrophages showed that PI3K, Src, and Syk activities are required for FcR-mediated phagocytosis but not for the FcR-dependent endocytosis of small immune complexes (79), but recent papers confirm that PI3K, Src, and Syk are commonly activated upon FcγR engagement, although other pathways are cell-type and Fc receptor-type dependent (80). However, the P-Rex1-dependent activation of Syk required the Rac-GEF activity, so is unlikely to be a major mechanism through which P-Rex1 controls phagocytosis. The same applied to Rac. Rac1 and Rac2 are required for both Fc receptor-dependent and integrin-dependent phagocytosis in macrophages (59). Human neutrophils express mostly Rac2, whereas murine neutrophils express both Rac1 and Rac2 equally. P-Rex1 is predominantly a Rac2-GEF but also activates the other Rac isoforms (3, 4), and we show here that P-Rex1 activates both Rac1 and Rac2 in response to fMLP, as well as Rac2 in response to IgG-opsonized beads. Unfortunately, we were unable to measure Rac1 activation by IgG-opsonized beads for technical reasons. However, like Syk, the activation of Rac by P-Rex1 required the Rac-GEF activity. Therefore, Rac-GEF activity independent pathways through which P-Rex1 controls phagocytosis remain to be identified in the future.

In conclusion, we have shown that neutrophil chemotaxis, ROS production and NET formation require the catalytic Rac-GEF activity of P-Rex1. In contrast, the clearance of *E. coli* bacteria during septic peritonitis, neutrophil bactericidal activity, and phagocytosis are controlled by P-Rex1 independently of its Rac-GEF activity, through mechanisms which require further study but do not seem to involve previously identified adaptor functions in GPCR trafficking or glucose homeostasis. Recent years have seen a growing interest in GEF activity-independent roles of Rac-GEFs, as they open new avenues for targeting deregulated Rac-GEFs in disease, and our study adds to the rapidly growing list of interesting GEF-activity independent roles for P-Rex1.

## Data Availability

The original contributions presented in the study are included in the article/**Supplementary Material**. Further inquiries can be directed to the corresponding author.

## Footnotes

1
**Abbreviations:** C5a, complement factor C5a anaphylatoxin. DMEM, Dulbecco’s modified Eagle medium. DOG, 2-deoxy glucose. DPBS, Dulbecco’s phosphate buffered saline. FAF, fatty-acid free. fMLP, f-Met-Leu-Phe, fMLF. GPCR, G protein-coupled receptor. GEF, guanine-nucleotide exchange factor. GM-CSF, granulocyte-macrophage colony-stimulating factor. HBSS, Hank’s balanced salt solution. KRB, Krebs Ringer buffer. LB, Luria broth. mfi, mean fluorescence intensity. Mmp9, gelatinase, metalloproteinase 9. MPO, myeloperoxidase. NETs, neutrophil extracellular traps. PMA, phorbol 12-myristate 13-acetate. P-Rex1, phosphatidylinositol 3,4,5-trisphosphate-dependent Rac exchanger 1, PREX1. PIP_3_, phosphatidylinositol 3,4,5-trisphosphate. PI3K, phosphoinositide 3-kinase. ROS, reactive oxygen species. SOD, superoxide dismutase. SDF1α, stromal cell-derived factor 1α. TNFα, tumor necrosis factor α.
