# Supplementary material for: P-Rex1 controls phagocytosis and the killing of bacteria by murine neutrophils independently of its catalytic activity
Source: Front Immunol. 2025 Sep 30;16:1591006. doi: 10.3389/fimmu.2025.1591006 (PMC12518277; doi:10.3389/fimmu.2025.1591006)
Supplement: Supplementary file 1 [file DataSheet1.pdf]

Supplementary Material

P-Rex1 controls phagocytosis and the killing of bacteria by murine neutrophils independently of its catalytic activity

Priota Islam [1], Julia Y. Chu [1,2], Stephen A. Chetwynd [1,3], Rachael Walker [4], Phillip T. Hawkins [1], Heidi C. E. Welch [1,*]

[1] Signalling Programme and [4] Flow Cytometry Facility, The Babraham Institute, Babraham Research Campus, Cambridge, UK

[2] Present address: CAMS Oxford Institute, Nuffield Department of Medicine, University of Oxford, Headington, UK

[3] Present address: PlaqueTec, Babraham Research Campus, Cambridge, UK

*** Correspondence:** Heidi Welch, Signalling Programme, The Babraham Institute, Babraham Research Campus, Cambridge CB22 3AT, UK. +44 (0)1223 496 596. heidi.welch@babraham.ac.uk

Contents:

Supplementary Figures 1-12

Supplementary Figure Legends

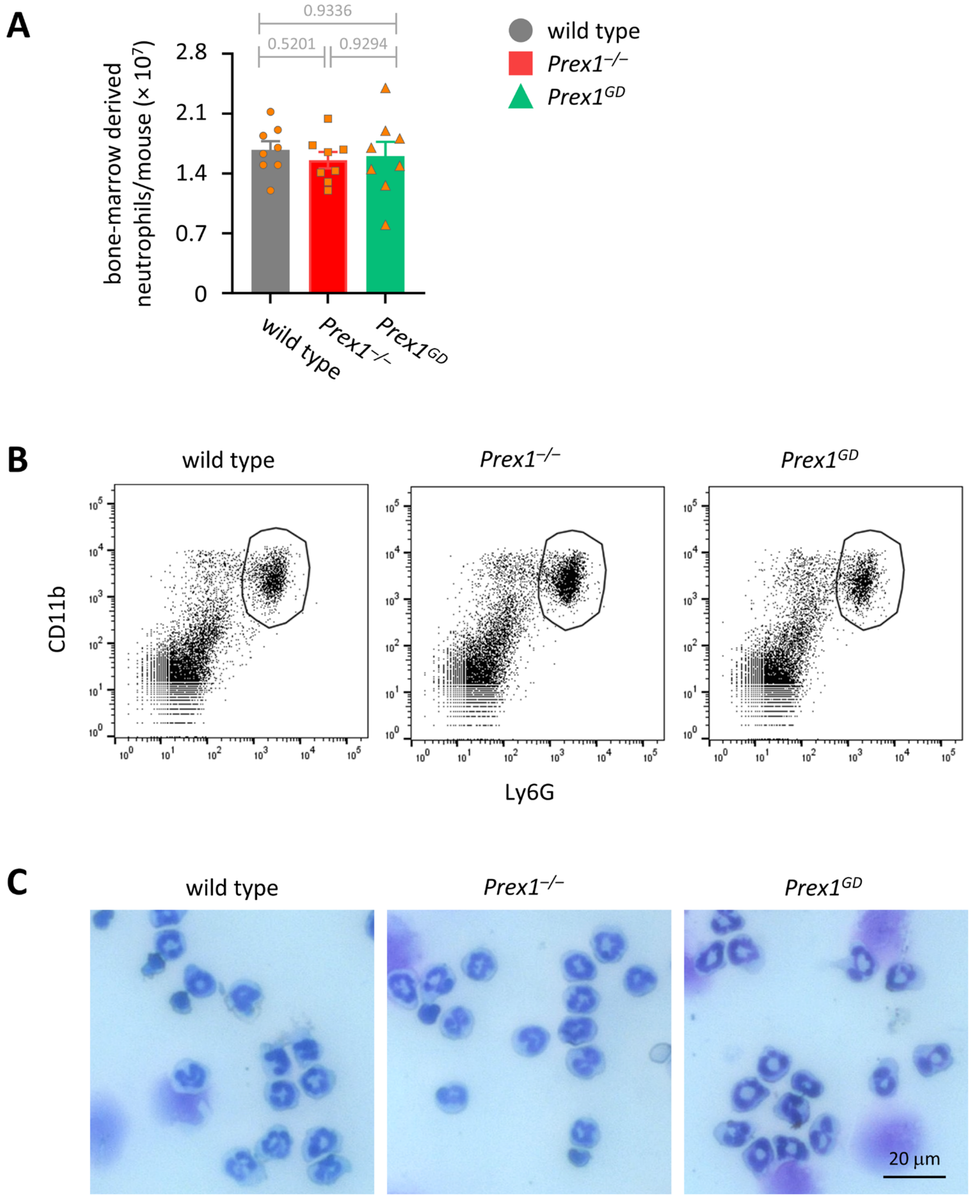

Supplementary Figure 1. P-Rex1 deficiency or catalytic inactivity do not affect neutrophil development. *Prex1*$^{-/-}$ (red squares), *Prex1*^{GD} (green triangles), and wild type mice (grey circles) were analysed for neutrophil numbers and maturity. **(A)** Numbers of mature neutrophils isolated from the bone marrow of 8–14-week-old mice. Data are mean ± SEM of 8 mice/genotype; each symbol represents one mouse. Statistics are one-way ANOVA with Tukey's multiple comparisons tests on raw data; grey p-values are non-significant. **(B)** Representative flow cytometry plots of bone marrow cells showing normal numbers and maturity of neutrophils as characterised by CD11b^{hi}, Ly6G^{hi} staining. **(C)** Representative cytospin images stained with Kwik-Diff depicting the characteristic doughnut-shaped nuclear morphology of purified, mature, bone-marrow derived neutrophils.

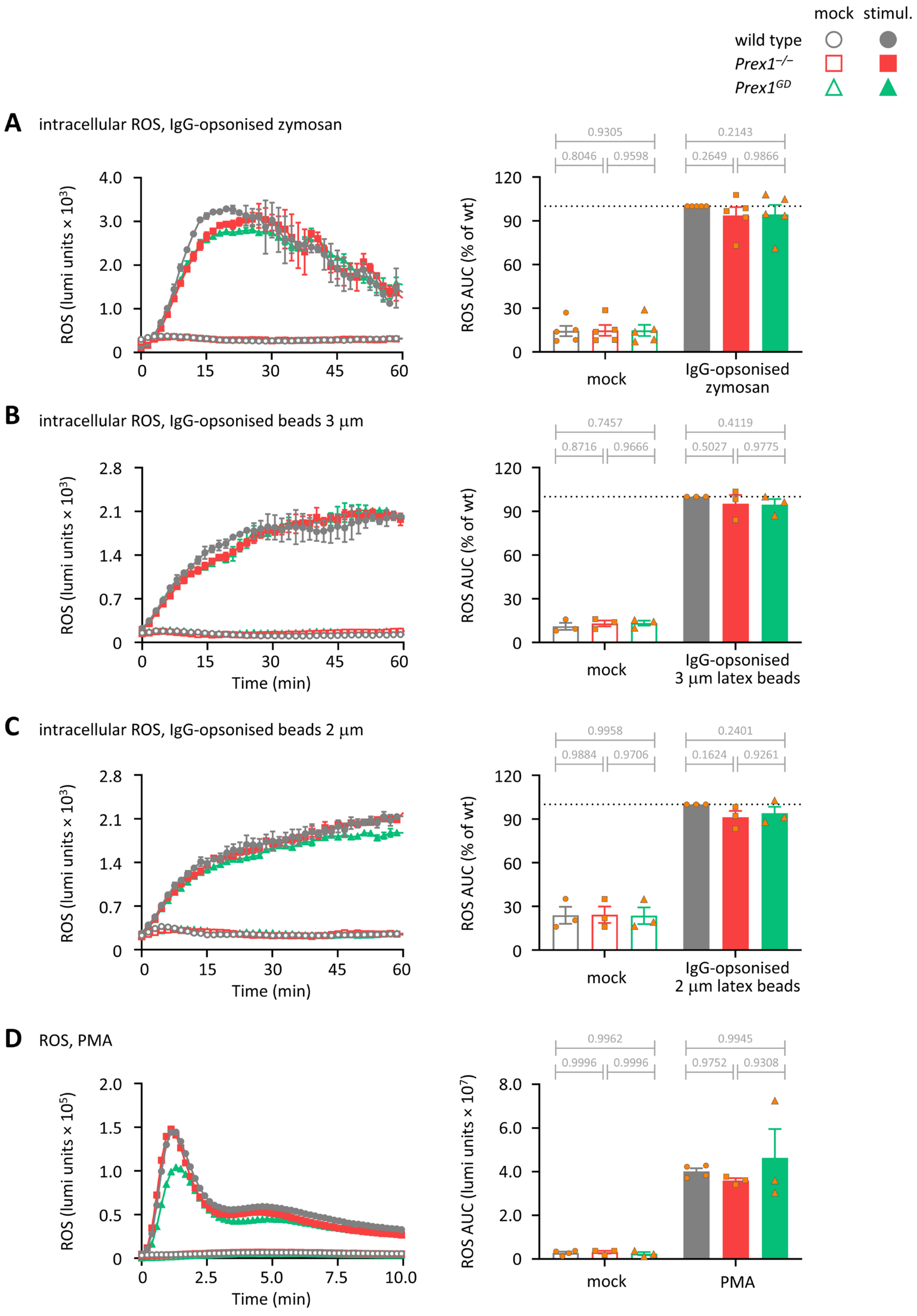

Supplementary Figure 2

Supplementary Figure 2. P-Rex1 does not regulate particle- or PMA-stimulated ROS production. (A-C) Particle-stimulated intracellular ROS production. Purified neutrophils from *Prex1*$^{-/-}$ (red squares), *Prex1*^{GD} (green triangles), and wild type mice (grey circles) were primed with 20 ng/ml TNFα and 50 ng/ml GM-CSF for 45 min in the presence of SOD and catalase to scavenge extracellular ROS. Cells were stimulated with (A) IgG-opsonised zymosan yeast particles at a ratio of 5 particles per neutrophil or with 3 μm diameter (B) or 2 μm diameter (C) IgG-opsonised latex beads at a ratio of 10 particles per neutrophil (filled symbols), or they were mock-stimulated (open symbols). Intracellular ROS production was measured in the presence of luminol, SOD, and catalase, but without HRP, by real-time chemiluminescence assay over 60 min. Left-hand panels show representative luminometer traces from one experiment; right-hand panels show the quantification as AUC, normalised to the particle-stimulated wild type (wt) for each experiment. Data are mean ± SEM of 3-5 independent experiments per particle type. **(D)** PMA-stimulated ROS production. Neutrophils were stimulated with 500 nM PMA (filled symbols), or mock-stimulated (open symbols), and ROS production was measured as in (A-C) except without SOD or catalase and with both luminol and HRP for total (extra- and intracellular) ROS, and over 10 min. Data are mean ± SEM of 3-4 independent experiments; each symbol represents the mean AUC from one experiment. Statistics in (A-D) are two-way ANOVA with Sidak's multiple comparisons tests on log-transformed raw data; grey p-values are non-significant

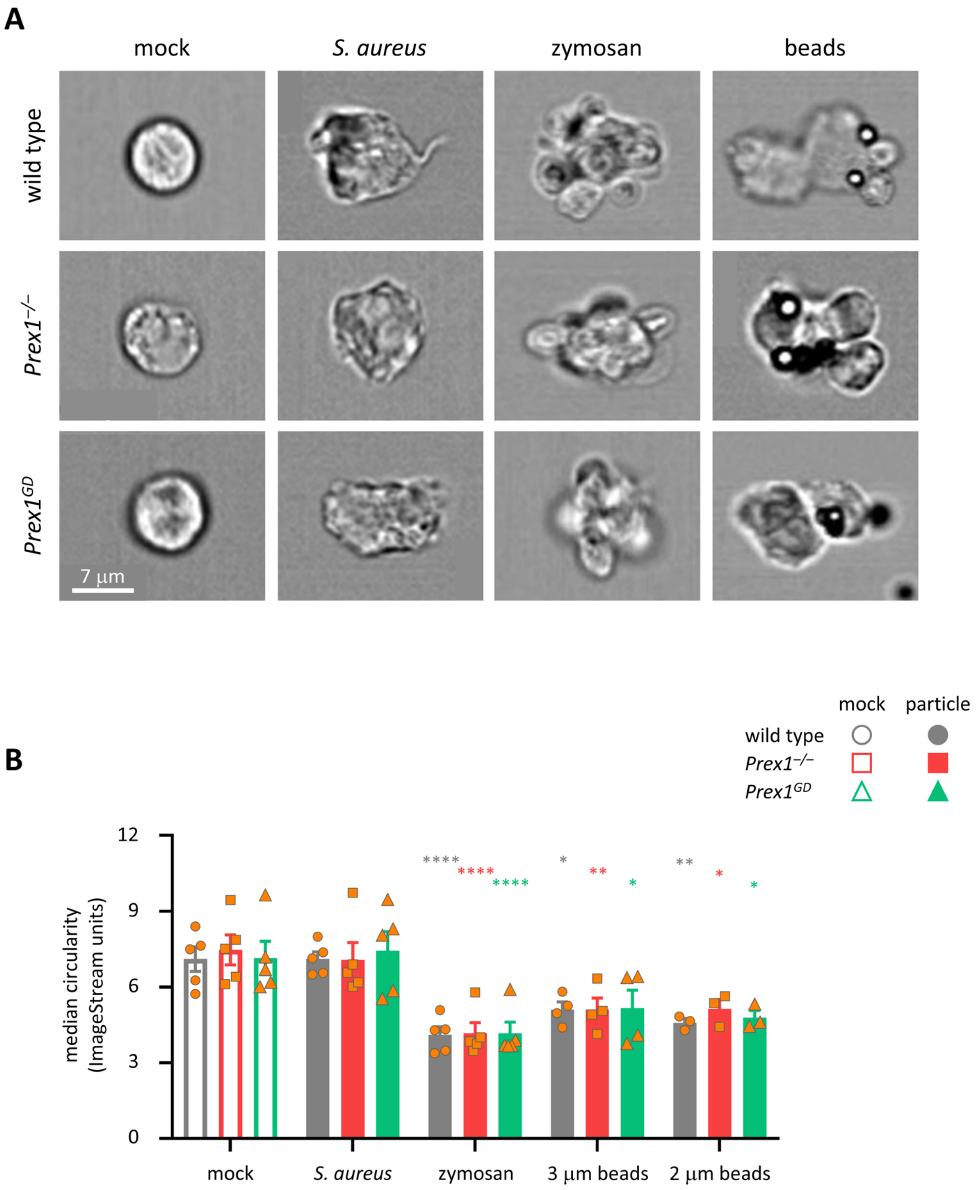

Supplementary Figure 3. P-Rex1 does not regulate cell-shape changes during particle-induced ROS production. Purified neutrophils from *Prex1*$^{-/-}$ (red squares), *Prex1*^{GD} (green triangles), and wild type mice (grey circles) were recovered after the intracellular ROS assays shown in Figure 2D and Supplementary Figure 2A-C, upon stimulation with serum-opsonised *S. aureus*, IgG-opsonised zymosan yeast particles, or 3 µm and 2 µm diameter IgG-opsonised latex beads (closed bars), or mock-stimulation (open bars). Cells were fixed and analysed by ImageStream imaging flow cytometry. **(A)** Representative brightfield images. **(B)** Median circularity of cells in focus in brightfield images quantified using the ImageStream software. Data are mean ± SEM of 3-5 independent experiments per particle type; statistics are two-way ANOVA with Sidak's multiple comparisons tests. Stars denote statistically significant differences between particle and mock stimulation; * denotes p<0.05, ** p<0.01, **** p<0.0001. There were no significant differences between genotypes.

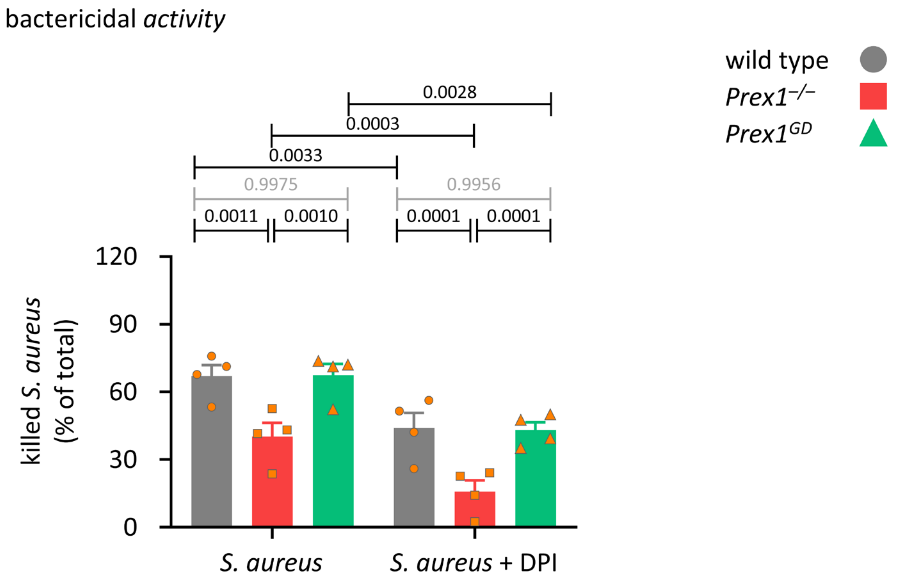

Supplementary Figure 4. P-Rex1 controls the ROS-independent part of bactericidal activity against *S. aureus*. Purified neutrophils from *Prex1*$^{-/-}$ (red squares), *Prex1*^{GD} (green triangles), and wild type mice (grey circles) were primed with 20 ng/ml TNFα and 50 ng/ml GM-CSF for 45 min before incubation with serum-opsonised *S. aureus* for 90 min at a ratio of 1.5 bacteria per neutrophil, as in Figure 2A, except that 10 μM diphenyleneiodonium (DPI) was added during priming and killing assay, or cells were mock-treated with vehicle control (1% DMSO), as indicated. The % killing of bacteria by live neutrophils compared to heat-killed controls is plotted. Data are mean ± SEM of 4 independent experiments; each symbol represents the mean of one experiment. Statistics are two-way ANOVA with Sidak's multiple comparisons test on square-root-transformed raw data; black p-values are significant, grey p-values non-significant.

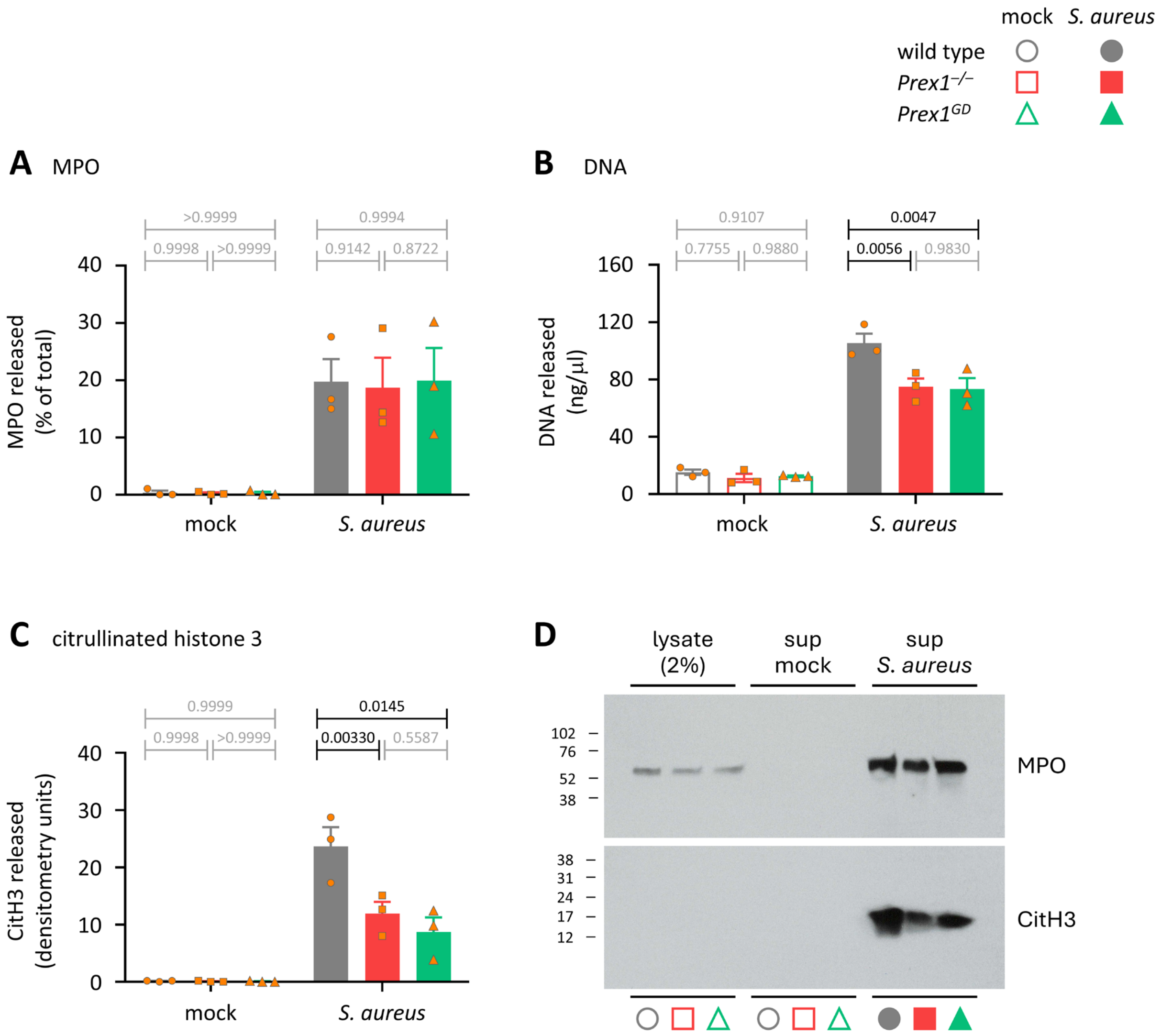

Supplementary Figure 5. P-Rex1 is required for the release of DNA and citrullinated histone 3, but not MPO, during NET formation in response to *S. aureus*. NET formation by purified neutrophils from *Prex1*$^{-/-}$ (red squares), *Prex1*^{GD} (green triangles), and wild type mice (grey circles) was assayed using NanoDrop spectrophotometry and western blotting. **(A-D)** Cells were incubated with serum-opsonised *S. aureus* for 3 h at 37 °C at a ratio of 12.5 bacteria per cell (closed symbols), or mock-stimulated for the same period of time in DPBS^{++++} (open symbols). A lysate of zero-time, mock-stimulated cells was prepared for comparison. After the incubation, cell supernatants were recovered, and (B) DNA release into the supernatant was assessed by NanoDrop, and the release of (A) MPO and (C) citrullinated histone 3 (CitH3) was assessed by western blotting. (D) Representative blots from one experiment are shown, with MPO and CitH3 from the same membrane for direct comparison. Data are mean ± SEM of 3 independent experiments; each symbol represents the mean of one experiment. Statistics are two-way ANOVA with Sidak’s multiple comparisons test; black p-values are significant, grey p-values non-significant.

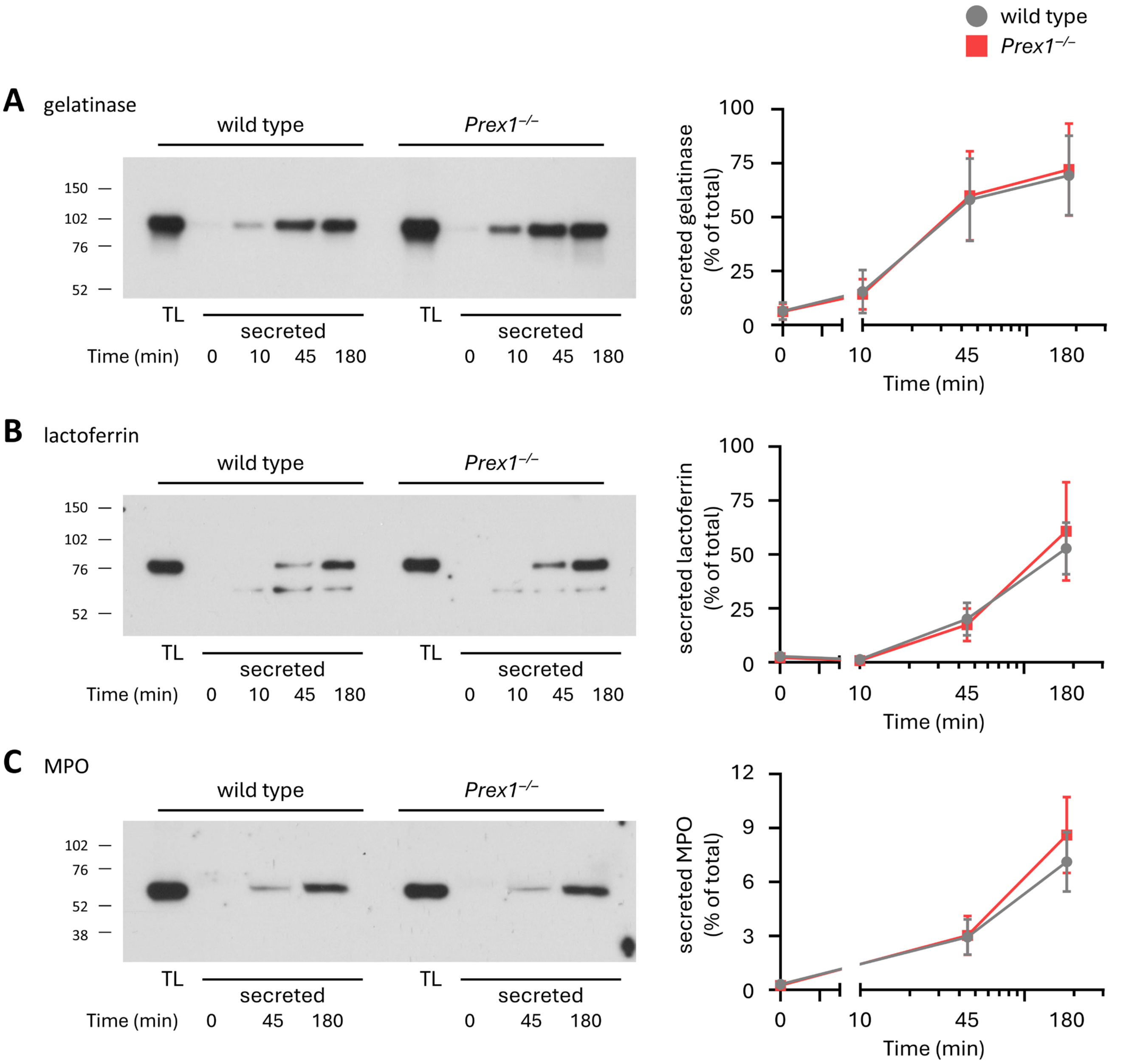

Supplementary Figure 6. P-Rex1 does not control neutrophil degranulation. Purified neutrophils from *Prex1*$^{-/-}$ (red squares) and wild type mice (grey circles) were stimulated with serum-opsonized *E. coli* (DH5α) for the indicated periods of time at a ratio of 12.5 bacteria per neutrophil. Cell supernatants containing secreted proteins were harvested at the time points indicated. A control cell pellet (TL) was prepared from neutrophils prior to stimulation with *E. coli*. Samples were analysed by Western blotting for the degranulation of gelatinase **(A)**, lactoferrin **(B)**, and MPO **(C)**. Quantification was done using Fiji densitometry, and secretion into the cell supernatant was calculated as % of the TL control for each protein. The ratios of TL to supernatant loaded in were 1:2.5 in (A, B) and 1:80 in (C). Data are mean ± SEM of 4 independent experiments. Statistics were two-way ANOVA with Sidak's multiple comparisons test on log-transformed raw data and showed no significant differences.

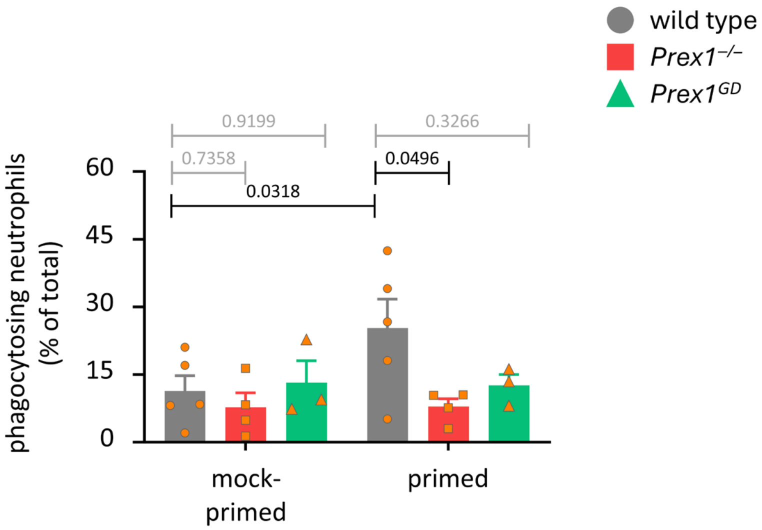

Supplementary Figure 7. P-Rex1 mediates the phagocytosis of IgG-opsonised latex beads in primed neutrophils. Purified neutrophils from *Prex1*$^{-/-}$ (red squares), *Prex1*^{GD} (green triangles), and wild type mice (grey circles) were primed with 20 ng/ml NFα and 50 ng/ml GM-CSF for 45 min, or were mock-primed, before being allowed to adhere to glass coverslips for 15 min, and stimulation with IgG-opsonized latex beads for 60 min at a ratio of 10 particles per neutrophil. Cells were stained, imaged, and image analysis performed as in Figure 4. Data are mean ± SEM of 3-5 independent experiments, each symbol represents the mean of one experiment. Statistics are two-way ANOVA with Sidak's multiple comparisons tests on square root-transformed raw data; black p-values are significant, grey p-values non-significant.

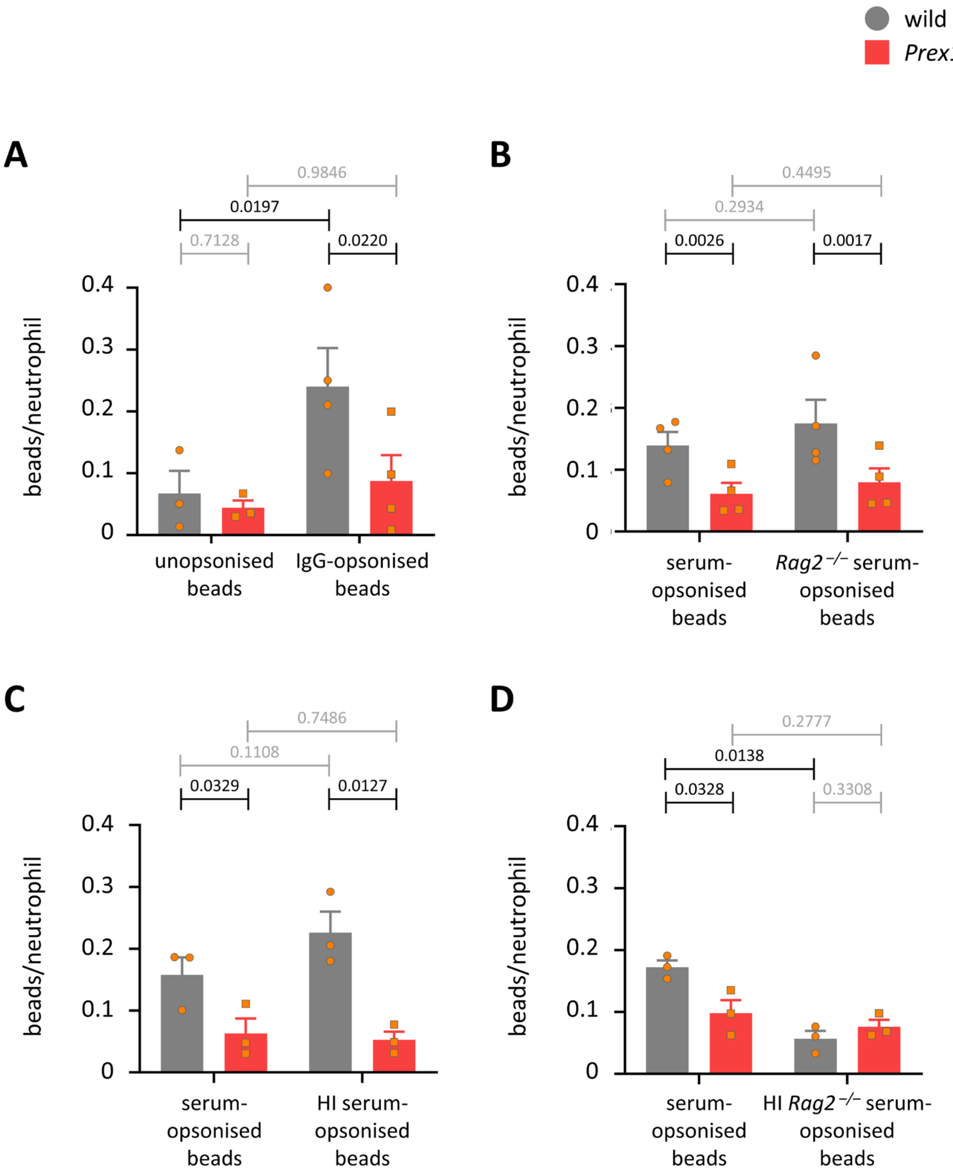

Supplementary Figure 8. P-Rex1 mediates both the integrin- and Fc receptor-dependent phagocytosis of latex beads. (A-D) Phagocytosis of latex beads. Data are from the same experiments as shown in Figure 5, measuring phagocytosis of latex beads by neutrophils from *Prex1*$^{-/-}$ (red squares) and wild type mice (grey circles) upon opsonisation of the beads with various reagents. Samples were analysed here for the number of beads phagocytosed per neutrophil; (A) comparing unopsonised and IgG-opsonized latex beads; (B) opsonisation with serum from wild type and *Rag2*$^{-/-}$ mice; (C) opsonisation with serum with or without prior heat-inactivation of complement factors; (D) opsonisation with serum from *Rag2*$^{-/-}$ mice with or without prior heat-inactivation of complement factors. Data are mean ± SEM of 3-4 independent experiments for each panel; each symbol represents the mean of one experiment. Statistics are two-way ANOVA with Sidak's multiple comparisons tests on square root-transformed raw data; black p-values are significant, grey p-values non-significant.

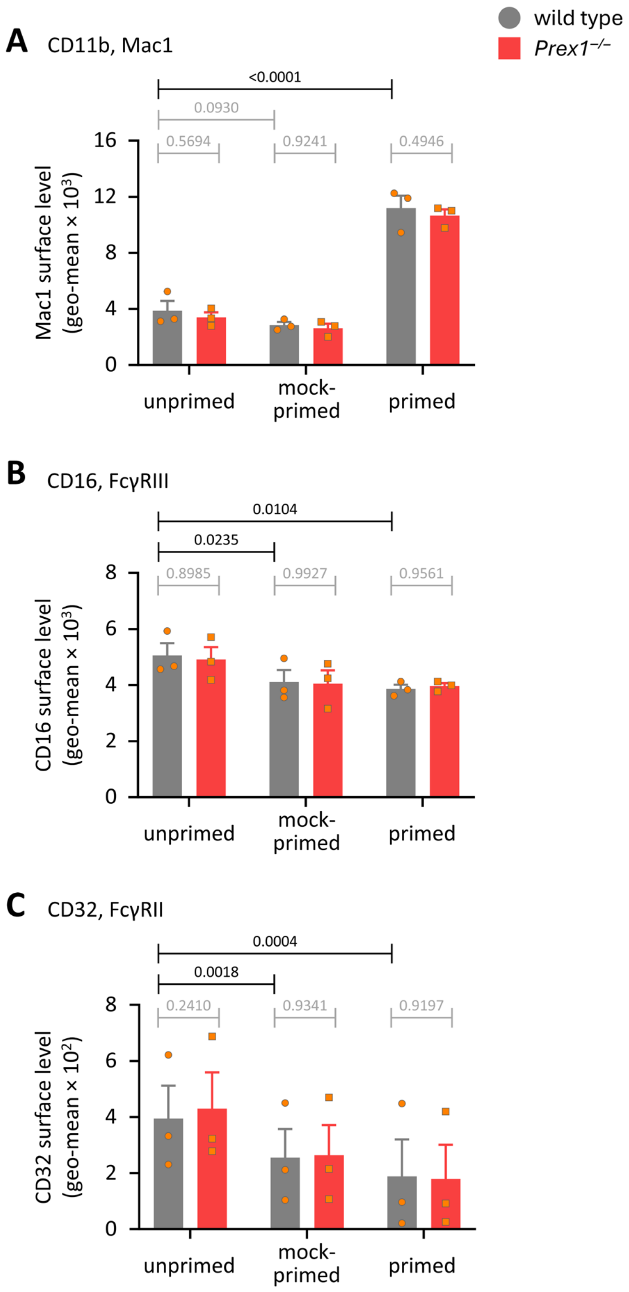

Supplementary Figure 9. P-Rex1 does not regulate the cell surface levels of adhesion receptors CD11b/Mac1, CD16/FcγRIII, or CD32/FcγRII. Bone marrow cells from *Prex1*$^{-/-}$ (red squares) and wild type mice (grey circles) were either kept on ice, or primed with TNFα and GM-CSF for 45 min, or mock-primed, before staining on ice for neutrophil marker Ly6G and for **(A)** CD11b/Mac1, **(B)** CD16/FcγRIII, or **(C)** CD32/FcγRII, and analysis by flow cytometry. The geo-mfi of receptor levels on the neutrophil surface were quantified using FlowJo. Data are mean ± SEM of 3 independent experiments for each panel; each symbol represents the mean of one experiment. Statistics are two-way ANOVA with Sidak's multiple comparisons tests; black p-values are significant, grey p-values non-significant.

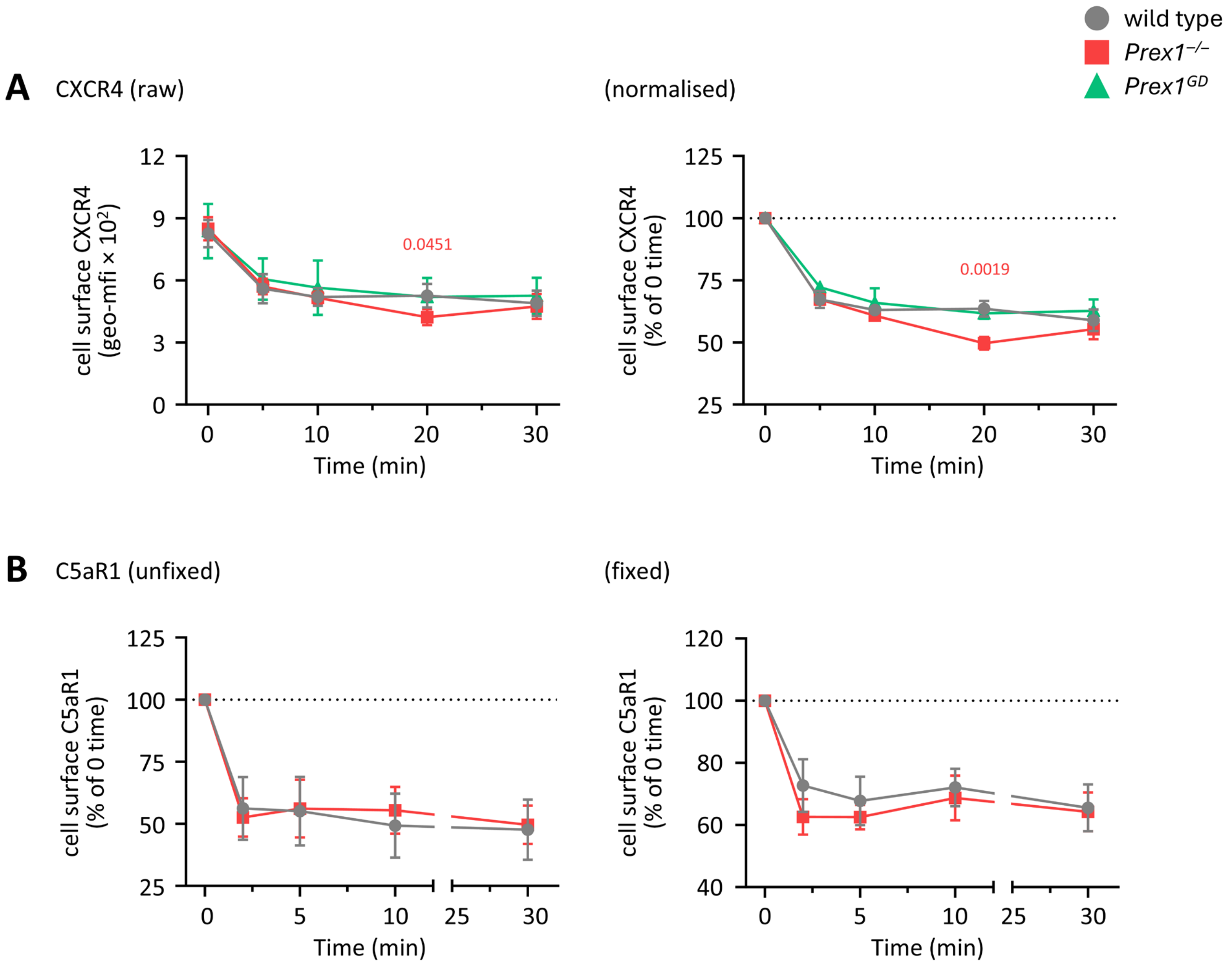

Supplementary Figure 10. P-Rex1 does not control the agonist-induced internalisation of CXCR4 or C5aR1. (A, B) Bone marrow cells from *Prex1*$^{-/-}$ (red squares), *Prex1*^{GD} (green triangles), and wild type mice (grey circles) were stimulated with (A) 100 nM SDF1α or (B) 50 nM C5a for the indicated periods of time, before staining on ice for neutrophil markers Ly6G and Mac1, and for (A) CXCR4 or (B) C5aR1, and analysis by flow cytometry. Neutrophils were identified by their Ly6G^{hi}/Cd11b^{hi} staining, and C5aR1 and CXCR4 cell surface levels (geo-mfi) were determined using FlowJo. In (A), data in the left-hand panel are raw data, data on the right are normalised to the 0-time control. In (B), data on the left are normalised to 0-time control, data on the right are cells fixed in paraformaldehyde prior to staining. Data are mean ± SEM of 4 independent experiments in (A) and 3 in (B). Statistics are two-way ANOVA with Sidak's multiple comparisons tests; red p-values show significant differences between wild type and *Prex1*$^{-/-}$.

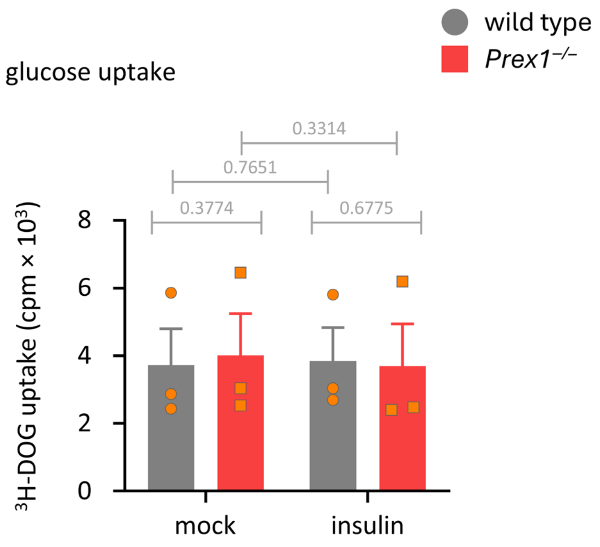

Supplementary Figure 11. P-Rex1 does not control glucose uptake in neutrophils. Purified neutrophils from *Prex1*$^{-/-}$ (red squares) and wild type mice (grey circles) neutrophils were stimulated with 200 nM insulin in the presence of 50 µM DOG and 0.25 µCi H^3-2-DOG for 30 min, or mock-stimulated, washed, and lysed. Uptake of DOG was measured by scintillation counting. Data are mean ± SEM of 3 independent experiments. Statistics are two-way ANOVA with Sidak's multiple comparisons tests; grey p-values are not significant.

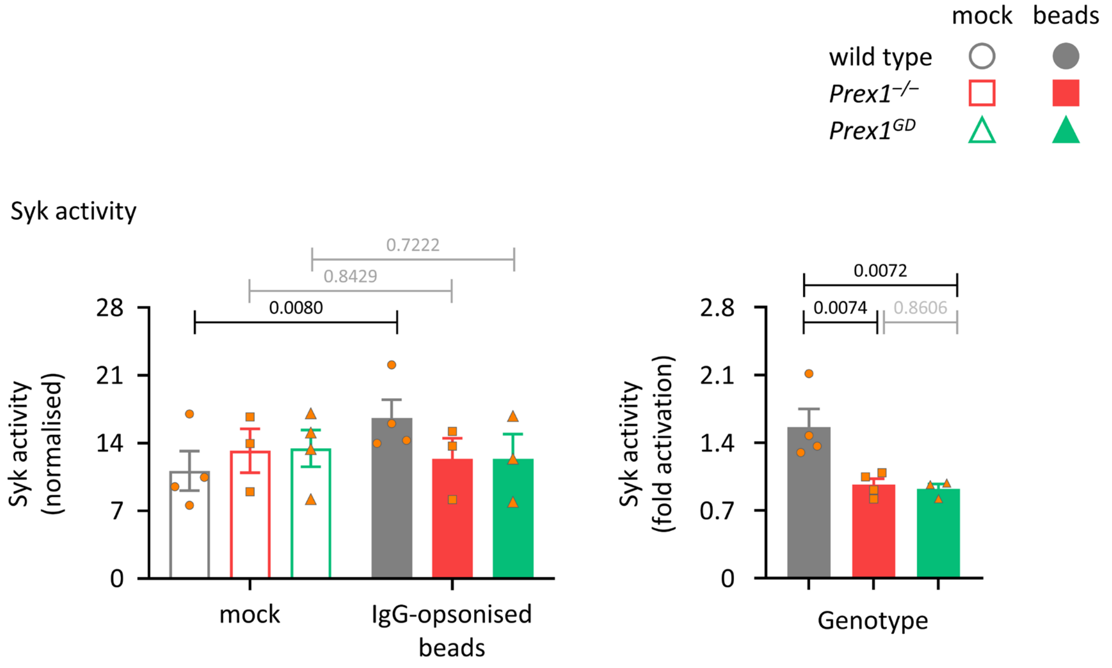

Supplementary Figure 12. P-Rex1 mediates the activation of Syk by IgG-opsonised latex beads through its Rac-GEF activity. Purified neutrophils from *Prex1*$^{-/-}$ (red squares), *Prex1*^{GD} (green triangles), and wild type mice (grey circles) were primed with TNFα and GM-CSF for 45 min before stimulation with IgG-opsonised latex beads for 30 min, or mock-stimulation. Total cell lysates were analysed by Western blotting for phospho-Syk, and blots were quantified as in Figure 6C. Data are mean ± SEM of 3 independent experiments. Statistics are two-way ANOVA with Sidak's multiple comparisons tests; black p-values are significant, grey p-values non-significant.
